# Transdiagnostic physiological correlates of unpredictable threat anticipation

**DOI:** 10.1016/j.ijchp.2026.100723

**Published:** 2026-09-14

**Authors:** Hannes Per Carsten, Kai Härpfer, Franziska Magdalena Kausche, Anja Riesel

**Affiliations:** Department of Psychology, University of Hamburg, Hamburg, Germany

**Keywords:** Anxiety, Obsessive-compulsive disorder, Uncertainty, Anticipatory anxiety, Unpredictable threat, Latent profile analysis

## Abstract

Responses to unpredictable threat have been proposed as a candidate mechanism in anxiety-related psychopathology, but clinical findings remain heterogeneous and often rely on single physiological outcomes. The present study examined whether physiological responses during threat anticipation map onto categorical and dimensional conceptualizations of psychopathology across the anxiety and obsessive-compulsive spectrum. In a transdiagnostic sample of *N* = 163 participants, patients with specific phobia, social anxiety disorder (SAD), and obsessive-compulsive disorder (OCD), as well as unaffected controls completed the NPU-threat test in order to measure responses to predictable and unpredictable threat. Physiological responding was assessed using startle reflex, skin conductance responses (SCR), and event-related potentials indexing early sensory processing (N1) and later attentional allocation (P3). Results indicated a group-based effect for SCR, such that patients with OCD showed enhanced autonomic responding during unpredictable threat anticipation. Dimensionally, anxious arousal was associated with reduced SCR during unpredictable threat and lower P3 amplitudes across conditions. Intolerance of uncertainty modulated startle particularly in controls, and anxious apprehension was associated with increased N1 amplitudes in OCD. Exploratory latent profile analysis identified three multisystem physiological response profiles. A threat-reactive profile characterized by increased N1 and both increased SCR and startle to unpredictable threat was particularly clinically enriched, showing higher clinical severity, greater representation of SAD and OCD, and elevated anxious apprehension and social anxiety symptoms. Together, findings suggest that threat anticipation in anxiety-related psychopathology is best understood as a multisystem and partly transdiagnostic process rather than as a single physiological marker of diagnostic status or unpredictable-threat sensitivity.

## Introduction

Anxiety disorders are highly prevalent (Baxter et al., 2013), debilitating, and account for a substantial share of the disease burden attributable to mental disorders, as measured by disability-adjusted life years (Baxter et al., 2014; Murray et al., 2012). At the same time, anxiety disorders are highly heterogeneous, with patients differing in symptom expression, severity, chronicity, comorbidity, and functional impairment (American Psychiatric Association, 2013). This heterogeneity complicates efforts to identify the neural and psychophysiological processes underlying different phenotypic expressions of anxiety and to translate these insights into targeted interventions (Cuthbert & Insel, 2013). Responses to unpredictable threat represent one candidate process implicated in anxiety-related psychopathology (Abend, 2023; Grupe & Nitschke, 2013; Rosen & Schulkin, 1998). The present study therefore examined how unpredictable threat anticipation varies across patients with different anxiety disorders and obsessive-compulsive disorder (OCD).

Neurobiologically informed models suggest that uncertainty plays a central role in the formation and maintenance of anxiety-related psychopathology. Specifically, anxiety-related psychopathology has been linked to elevated threat expectancies (Grupe & Nitschke, 2013), impaired safety inference in ambiguous contexts (Brosschot et al., 2017, 2018), and a bias towards threat-based responding under uncertainty (Freeston & Komes, 2023; Van den Bergh et al., 2021). In addition, elevated levels of self-reported trait intolerance of uncertainty have been consistently observed in anxiety-related psychopathology (Boelen & Reijntjes, 2009; Carleton et al., 2013; Gentes & Ruscio, 2011; Holaway et al., 2006; Mahoney & McEvoy, 2012; McEvoy et al., 2019; Tolin et al., 2003).

Experimental approaches have operationalized uncertainty by varying the predictability of aversive events, most prominently in the NPU-threat test, an instructed threat-of-shock paradigm developed by Schmitz and Grillon (2012). Here, the degree of predictability varies directly, which distinguishes responses to predictable from responses to unpredictable threat. Specifically, in the predictable condition (P), participants receive a highly predictable aversive stimulus whose occurrence is signaled by a cue. In contrast, participants receive unsignaled aversive stimuli in the unpredictable condition (U), whereas in the neutral condition (N), participants never receive an aversive stimulus (Schmitz & Grillon, 2012). The increase in responding from neutral to unpredictable threat is typically interpreted as anxiety potentiation, whereas increased responding to predictable threat is interpreted as fear potentiation. Previous studies have used the startle reflex to assess defensive responding (Blumenthal et al., 2005) in the NPU-threat test (Carsten et al., 2022, 2023; Gorka et al., 2017; Grillon et al., 2017; Hoge et al., 2024; Nelson et al., 2016; Nelson & Shankman, 2011; Schmitz & Grillon, 2012). Complementary electroencephalography enables studying the attentional dynamics during threat anticipation. Specifically, the event-related potential component N1 locked to acoustic startle probes indexes early sensory processing of the probes, whereas the P3 component reflects later attentional allocation. In a more aversive context, a general neural response pattern involves increased startle (Schmitz & Grillon, 2012), increased probe-locked N1 (Carsten et al., 2023; Nelson et al., 2015) and decreased probe-locked P3 (Ferry & Nelson, 2020; MacNamara & Barley, 2018; Nelson et al., 2015). Here, a less positive (i.e., suppressed) P3 is interpreted as the allocation of attentional resources to the threat cue rather than to the startle probe.

Clinical studies using the NPU-threat test suggest that anxiety-related psychopathology might be associated with alterations in unpredictable threat anticipation, but the pattern of findings across diagnostic groups is inconsistent. In a sample of patients with mixed anxiety disorders, increased startle during unpredictable but not predictable threat was found (Hoge et al., 2024). Heightened startle potentiation during unpredictable threat anticipation has been reported in patients with panic disorder and posttraumatic stress disorder (PTSD; Grillon et al., 2008, 2009) with subsequent findings implicating that panic attacks might be particularly relevant for increased responding to unpredictable threat in patients with generalized anxiety disorder (GAD) and/or social anxiety disorder (SAD; Grillon et al., 2017). However, other studies challenge this association of heightened unpredictable-threat responding in GAD in combination with panic attacks. For instance, Gorka et al. (2017) found elevated startle potentiation to unpredictable threat in more fear-based disorders, including specific phobia. Next, while some studies linked panic disorder to increased responses to unpredictable threat (Grillon et al., 2008; Shankman et al., 2013), other studies found contradictory response patterns (Gorka et al., 2014; Stevens et al., 2018). Additionally, heightened reactivity to unpredictable threat has also been observed in externalizing psychopathology such as alcohol use disorder (Gorka et al., 2020; Gorka & Shankman, 2017), whereas blunted or reduced reactivity has been reported in relation to greater PTSD symptom severity (Lieberman et al., 2020) and comorbid panic disorder/PTSD (Katz et al., 2018). Further, previous research has not covered the breadth of the internalizing spectrum with patients with OCD being underrepresented in this field. Thus, although clinical studies using the NPU-threat test support the relevance of unpredictable threat anticipation for anxiety-related psychopathology, the inconsistent result pattern across diagnostic groups suggests substantial variability in effects. As outlined above, prior clinical NPU studies have typically relied on single outcome measures, most commonly startle potentiation, and on comparing responses to unpredictable threat anticipation in diagnostic groups with healthy controls. Thus, it remains unclear whether alterations in unpredictable threat anticipation generalize across response systems, including defensive reflex modulation, early sensory processing, and attentional allocation. Moreover, because anxiety disorders are heterogeneous and highly comorbid, diagnostic group comparisons may obscure clinically meaningful variation in symptom severity and dimensional traits (Cuthbert, 2015; Cuthbert & Insel, 2013; Krueger & Markon, 2006).

To map anxiety-related psychopathology, two integrative frameworks informed the research question of the present study, the framework of anxiety dimensions (Sharp et al., 2015; Snyder et al., 2023) and the spectrum from circumscribed fear to anxious misery (Lang et al., 2016). First, the dimensional and commonly shared decomposition of anxiety proposes two anxiety dimensions, which are considered phenomenologically and neurally distinguishable (Cox et al., 2010; Heller et al., 1997; Sharp et al., 2015; Snyder et al., 2023). Anxious apprehension grasps the tendency for verbal, future-oriented rumination and worry. Anxious arousal captures the propensity to experience symptoms of physiological hyperarousal when confronted with mild stressors (Clark & Watson, 1991; Sharp et al., 2015; Watson et al., 1995). While the evidence for anxious apprehension modulating responses to temporally unpredictable threat is scarce and inconsistent (Nelson & Shankman, 2011; Carsten et al., 2023; Rutherford et al., 2020), self-report anxious arousal has not yet been empirically linked to threat anticipation in clinical samples (Carsten et al., 2023). Prior studies on dimensional traits have linked intolerance of uncertainty to defensive responding in anticipation of temporally unpredictable threat (Nelson et al., 2016; Nelson & Shankman, 2011), although the evidence is mixed (Bennett et al., 2018; Lieberman et al., 2016; Morriss et al., 2020, 2022; Papenfuss et al., 2021) and findings might be specific to lower probability of threat (Carsten et al., 2022; Chin et al., 2016). Taken together, these findings suggest that individual difference constructs are promising candidates for explaining variance in threat anticipation, but existing evidence remains limited, inconsistent, and largely based on few clinical samples and single-outcome studies. Second, beyond symptom-level dimensions, anxiety disorder categories themselves may also be organized along a broader clinical spectrum from circumscribed fear to anxious misery. As such, Lang et al. (2016) mapped anxiety disorders along a gradient from circumscribed fear, such as specific phobia, through more generalized psychopathology, such as SAD, to “anxious misery”, including OCD and GAD. Movement toward the anxious misery pole was characterized by greater comorbidity, chronicity, functional impairment, and physiological hyporeactivity. These data were derived from disorder-specific aversive imagery paradigms (McTeague & Lang, 2012), in which threat predictability was not directly manipulated. Thus, it remains unclear whether the circumscribed fear-anxious misery spectrum also maps onto responses to predictable and unpredictable threat.

Taken together, prior research suggests that responses to unpredictable threat are relevant to anxiety-related psychopathology, but the multimodal physiological underpinnings as well as the clinical and potentially transdiagnostic pattern of this association remain unclear due to mixed prior findings. On the one hand, the spectrum from circumscribed fear to anxious misery suggests that anxiety disorder categories may differ in general physiological reactivity, with circumscribed fear characterized by heightened defensive responding and anxious misery by blunted responding (Lang et al., 2016). On the other hand, neurobiologically informed models of uncertainty and clinical NPU findings suggest that more generalized, impairing, and comorbid psychopathology may be associated with heightened sensitivity to unpredictable threat (Freeston & Komes, 2023; Grillon et al., 2008; Grupe & Nitschke, 2013; Hoge et al., 2024; Van den Bergh et al., 2021). These two patterns are not mutually exclusive: individuals closer to the anxious-misery pole may show reduced overall physiological reactivity while still displaying stronger condition-specific modulation by unpredictable threat. The principal research question was whether physiological reactivity and sensitivity to unpredictable threat across multiple response systems map systematically onto categorical and dimensional variation in anxiety-related psychopathology. To address the categorical component of this question we examined whether diagnostic groups ordered along the circumscribed fear-anxious misery spectrum differ in both general physiological reactivity and sensitivity to unpredictable threat across startle, SCR, N1, and P3 responses. In addition, we tested whether dimensional constructs of anxiety-related psychopathology can explain variation in responding to threat anticipation beyond the categorical diagnosis. Specifically, we examined self-reported intolerance of uncertainty, anxious apprehension, anxious arousal, and negative affect as predictors of physiological reactivity and sensitivity to unpredictable threat. We preregistered three hypotheses (https://osf.io/n9qxp): First, we expected general physiological reactivity to decrease with proximity to the anxious-misery pole, such that responses would be strongest in unaffected controls, followed by specific phobia, SAD, and OCD (H1: unaffected controls > specific phobia > SAD = OCD). Second, we expected sensitivity to unpredictable threat to increase along the same spectrum (H2: unaffected controls < specific phobia < SAD = OCD). Third, we expected dimensional constructs of anxiety-related psychopathology to account for individual differences in physiological reactivity and unpredictable-threat sensitivity beyond diagnostic categories (H3).

## Methods

### Participants

We recruited 163 participants in four groups matched for age, gender, and years of education. The recruited clinical groups included patients with specific phobia, SAD, OCD, and unaffected controls without the respective mental disorders. Participants’ age ranged from 18 to 63 (Table 1). Five participants were excluded due to premature termination of the experiment (*n* = 3) and hardware or software errors (*n* = 2), leaving a total sample of *N* = 158 participants. The sample size was determined by a power analysis for a different research question within the broader project (Härpfer et al., 2025). An a priori sensitivity analysis was conducted using Monte Carlo simulations to estimate the smallest detectable standardized effects in a two-level regression model given the planned sample size of *N* = 160 (Arend & Schäfer, 2019; Green & Macleod, 2016). For the effects of diagnostic group, and the interaction of NPU condition × group, default medium effect sizes of *d* = 0.50 were assumed. Following this power simulation for the aggregated EEG data (i.e., 6 observations per subject), the likelihood to detect the interaction was 95.30% (95% CI [93.80, 96.53]), for the startle data (in single trial analyses i.e., 48 observations per subject) the likelihood to detect the interaction was 99.90% (95% CI [99.44, 100.00], https://osf.io/n9qxp).

**Table 1:** Demographic and clinical characteristics of the total sample and subgroups.

	Full Sample (*N* = 158)	Control (*n* = 42)	PHOB (*n* = 40)	SAD (*n* = 39)	OCD (*n* = 37)	Group differences
	Mean (*SD*)	Mean (*SD*)	Mean (*SD*)	Mean (*SD*)	Mean (*SD*)	*F*- or χ²- Statistic
**Demographics**						
Age	28.63 (8.32)	29.19 (8.22)	27.73 (9.73)	28.72 (7.68)	28.92 (7.64)	*F*(3, 154) = 0.23, *p* = .872
Gender %	74.05 f 25.95 m	71.43 f 28.57 m	77.50 f 22.50 m	74.36 f 25.64 m	72.97 f 27.03 m	χ²(3) = 0.42, *p* = .936
Education	12.20 (1.02)	12.21 (0.93)	12.18 (1.01)	12.26 (1.12)	12.16 (1.07)	*F*(3, 154) = 0.07, *p* = .978
% Psychotropic Medication	10.78	0	5.26	18.18	32.14	χ²(3) = 14.41, *p* = .002
**Clinical**						
Diagnoses	1.83 (1.63)	0.19 (0.46)	1.88 (1.29)	2.69 (1.47)	2.73 (1.58)	*F*(3, 154) = 36.10, *p* < .001 Control < PHOB < SAD = OCD
CGI	3.71 (1.40)	1.98 (0.61)	3.80 (0.99)	4.58 (1.03)	4.62 (0.89)	*F*(3, 152) = 76.45, *p* < .001 Control < PHOB < SAD = OCD
GAF	69.55 (17.37)	86.39 (14.68)	75.40 (11.41)	58.34 (11.44)	55.62 (9.26)	*F*(3, 153) = 58.93, *p* < .001 Control > PHOB > SAD = OCD
IUS-12	31.10 (10.34)	24.93 (7.71)	28.45 (10.15)	38.69 (7.62)	32.97 (10.37)	*F*(3, 154) = 17.47, *p* < .001 Control = PHOB < OCD < SAD
PSWQ	50.82 (13.67)	40.24 (11.20)	48.68 (13.73)	57.77 (9.05)	57.81 (11.96)	*F*(3, 154) = 21.22, *p* < .001 Control < PHOB < SAD = OCD
MASQ	25.87 (8.37)	21.86 (5.76)	24.55 (8.11)	28.13 (7.93)	29.46 (9.54)	*F*(3, 154) = 7.62, *p* < .001 Control < SAD/OCD^†^
STAI-T	44.93 (13.06)	35.57 (8.33)	40.05 (12.36)	53.36 (10.04)	51.95 (11.69)	*F*(3, 154) = 26.98, *p* < .001 Control = PHOB < SAD = OCD
STAI-S	39.24 (11.52)	32.66 (6.10)	35.00 (9.12)	45.53 (10.37)	44.68 (13.72)	*F*(3, 152) = 16.66, *p* < .001 Control = PHOB < SAD = OCD
LSAS	42.44 (31.79)	20.98 (16.99)	31.59 (23.93)	71.46 (28.76)	47.65 (31.13)	*F*(3, 153) = 29.51, *p* < .001 Control = PHOB < OCD < SAD
SMSP	6.30 (7.51)	1.95 (3.73)	8.64 (7.81)	6.54 (7.39)	8.41 (8.56)	*F*(3, 152) = 7.72, *p* < .001 Control < PHOB = SAD = OCD
OCI-R	13.43 (12.50)	5.98 (5.42)	9.13 (10.00)	11.54 (8.86)	28.54 (11.43)	*F*(3, 154) = 46.84, *p* < .001 Control = PHOB = SAD < OCD
AUDIT	4.04 (3.14)	3.74 (3.10)	4.45 (2.90)	3.67 (2.82)	4.35 (3.80)	*F*(3, 154) = 0.66, *p* = .579 Control = PHOB = SAD = OCD
BDI-II	11.32 (11.58)	4.27 (5.27)	7.88 (9.53)	16.97 (12.51)	17.08 (12.23)	*F*(3, 154) = 16.17, *p* < .001 Control = PHOB < SAD = OCD

*Note*. Control = control group; PHOB = specific phobia group; SAD = social anxiety disorder group; OCD = obsessive-compulsive disorder group; CGI = Clinical Global Impression Scale; GAF = Global Assessment of Functioning; AUDIT = Alcohol Use Disorders Identification Test; BDI-II = Beck Depression Inventory-II; IUS-12 = Intolerance of Uncertainty Scale-12; LSAS = Liebowitz Social Anxiety Scale-Self-Report; MASQ = Mood and Anxiety Symptom Questionnaire-Anxious Arousal; OCI-R = Obsessive-Compulsive Inventory-Revised; PSWQ = Penn State Worry Questionnaire; SMSP = Severity Measure for Specific Phobia; STAI-S = State–Trait Anxiety Inventory-State; STAI-T = State-Trait Anxiety Inventory-Trait. ^†^All other pairwise comparisons were nonsignificant.

Patients were recruited via the outpatient units of the University of Hamburg, student recruiting systems of the university, advertisement on the campus and social media, and platforms for part-time jobs. Participants received course credit or monetary compensation for the time of their participation. Inclusion criteria encompassed age (18-65 years), normal or corrected-to-normal vision, and the ability to give informed consent (assessed via self-report). Exclusion criteria were assessed via self-report and included a history of any neurological disorder, use of benzodiazepines (within the last week), neuroleptic medication (within the last three months). Exclusion criteria were assessed via the Structured Clinical Interview for DSM-5 Disorders-Clinician Version (SCID-5-CV; Beesdo-Baum et al., 2019) and included substance-related disorder (lifetime), schizophrenia spectrum and other psychotic disorders (lifetime), and manic episode (current). For the unaffected control group, exclusion criteria further encompassed lifetime diagnoses of disorders specifying the other groups, i.e., specific phobia, SAD, and OCD. Table 1 shows demographic and clinical characteristics of the full sample and groups.

### Clinical measures

On the day of the laboratory assessment, participants completed the following questionnaires: Intolerance of uncertainty was measured using the short form of the Intolerance of Uncertainty Scale (IUS; 12 items, 5-point Likert scale 1-5; α = .90; Carleton et al., 2007). Worry was measured by the Penn State Worry Questionnaire (PSWQ; 16 items, 5-point Likert scale 1-5; α = .94; Glöckner-Rist & Rist, 2014; Meyer et al., 1990). Anxious arousal was measured by the respective subscale of the Mood and Anxiety Symptom Questionnaire (MASQ; 17 items, 5-point Likert scale 1-5; α = .88; Clark & Watson, 1991; Watson et al., 1995). Depressive symptoms and negative affect were measured using the Beck Depression Inventory-II (BDI-II; 21 items, 4-point Likert scale 0-3; α = .95; Beck et al., 1996; Hautzinger et al., 2006). Further questionnaire data were acquired in order to describe the symptomatology of the sample. These questionnaires are not part of the confirmatory hypothesis testing but are included in exploratory analyses. State and trait anxiety were measured using the respective subscales of the State-Trait-Anxiety Inventory (STAI-T (α = .95) and STAI-S (α = .95); each 20 items; 4-point Likert scale 1-4; Laux, 1981; Spielberger et al., 1983). Social phobia symptoms were measured using the self-report version of the Liebowitz Social Anxiety Scale (LSAS; 24 items, 4-point Likert scale 0-3; α = .98; Liebowitz, 1987; Stangier & Heidenreich, 2005). Specific phobia symptoms were measured using the Severity Measure for Specific Phobia (SMSP; 10 items, 5-point Likert scale 0-4; α = .86; Craske et al., 2013). Obsessive-compulsive symptoms were measured using the Obsessive-Compulsive Inventory Revised (OCI-R; 18 items, 5-point Likert scale 0-4; α = .91; Foa et al., 2002; Gönner et al., 2007). Alcohol consumption was measured using the Alcohol Use Disorder Identification Test (AUDIT; 10 items, 5-point Likert scale 0–4; α = .66; Babor et al., 2001). Trained psychologists performed the diagnostics using the Structured Clinical Interview for DSM-5 Disorders-Clinician Version (SCID-5-CV; Beesdo-Baum et al., 2019). The overall severity was assessed via the Clinical Global Impression Scale (CGI; Guy, 1976), and overall functioning was assessed via the Global Assessment of Functioning (GAF; American Psychiatric Association, 2000).

### Procedure

The local ethics committee at the University of Hamburg approved the study procedure (AZ: 2021_351) to be in accordance with the Declaration of Helsinki (World Medical Association, 2013). Prior to the laboratory assessment, participants were screened for eligibility in a phone interview. Upon arrival in the laboratory, participants received verbal and written information about the objectives and methods of the study and gave written informed consent. After completing questionnaires, participants were prepared for the psychophysiological measurement and performed the NPU-threat test as a part of a larger project.

### Task and stimuli

Participants were seated in a dimly lit, electrically shielded cabin at a viewing distance of approx. 60 cm in front of a 19-inch monitor with a resolution of 1920 × 1080 pixels and a refresh rate of 120 Hz. To assess responses during unpredictable threat anticipation, a countdown version of the NPU-threat test (Nelson & Shankman, 2011; Schmitz & Grillon, 2012) was used. In the P condition, participants received a highly predictable, aversive electrotactile stimulation signaled by a cue (the “1″ of a countdown). In contrast, participants received unsignaled electrotactile stimulation in the U condition, whereas in the N condition, participants never received an aversive stimulus. Each condition occurred twice, resulting in two possible orders (PNUPNU or UNPUNP) that were counterbalanced across participants. A black screen stating the current condition served as the interstimulus interval (12 ± 3 s) and a numerical countdown counting from five to one served as the threat cue (1 s per digit). Each condition included nine cues (i.e., five interstimulus intervals and four countdowns) and a black screen (10 s) that separated the conditions. Four aversive stimuli occurred per condition (i.e., P and U). In the P condition, the aversive stimulus occurred when the countdown reached “1”, in the U condition the aversive stimulus could occur at any time during the countdown (4.2 ± 0.5 s) and interstimulus interval (5.5 ± 2.5 s). Startle probes were delivered via headphones (Sony MDR-ZX110) to elicit the startle reflex in 100% of the interstimulus intervals and countdowns. The startle probes consisted of 95 dB bursts of white noise with a duration of 50 ms (rise/fall time < 1 ms). Prior to the experiment, participants received 16 acoustic startle probes to allow an initial startle habituation (Blumenthal et al., 2005). A total of 64 startle probes were delivered per participant. The aversive stimulus consisted of an individually calibrated electrotactile stimulation. The calibration followed a staircase procedure (Lonsdorf et al., 2017), in which the participant subjectively rated the stimulus from 0 (not aversive) to 10 (extremely aversive, not endurable). The intensity of the aversive stimulus was increased stepwise to a subjective rating of 7–8 (*M* = 2.59 mA, *SD* = 1.48, range 0.30 - 13.00). During the calibration procedure, a minimum of 10 electrotactile stimuli were presented, to decrease habituation effects during the task.

### Startle recording and processing

Two Ag/AgCl electrodes with a sensor diameter of 6 mm were placed below the right eyelid to measure the surface electromyography of the eyeblink component of the startle reflex. A ground electrode was placed on the right infraorbital triangle. Data were recorded with a sampling rate of 1000 Hz. Preprocessing involved a band-pass filter on raw signal (28 - 500 Hz, 24 dB/octave roll-off), rectification, baseline correction (50 ms prior to startle probe onset), signal integration (9 ms moving average) and segmentation (−500 to 250 ms relative to startle probe). Data were then automatically scored using a custom-made algorithm in Brain Vision Analyzer 2.3. This involved the identification of missings, i.e., segments with spontaneous blinks in the baseline period (−50 to 20 ms relative to startle probe). If the data in this segment deviated by 4 *SD* from one of two 200 ms baseline periods (−500 to −300 ms and/or −300 to −100 ms relative to startle probe), the segment was scored as missing (*M* = 13.50%, *SD* = 7.98%). We implemented two baseline periods to decrease the probability that spontaneous blinks were included in one baseline period and thus missings in the refractory time window (i.e., −50 to 20 ms relative to startle probe) would not be detected based on the 4 *SD* deviation criterion. In segments without missings, startle blink amplitudes were scored using the EMG Onset Search solution implemented in Brain Vision Analyzer 2.3. As preregistered, the onset of a startle response was scored if there was a 3 *SD* deviation from the −50 to 20 ms baseline in a 20 - 120 ms onset window relative to the startle probe. A subsequent peak was scored if it occurred within 25 - 150 ms relative to the startle probe. If the response threshold of 3 *SD* was not met, the segment was scored as a nonresponse and included in the analysis (*M* = 5.11%, *SD* = 9.68%). To calculate the interrater reliability of this automatic scoring, we compared it with a manual scoring of an experienced rater blinded to the experimental conditions (cf. Carsten et al., 2023). Intraclass correlations were calculated between the automatic scoring and manual scoring on trial level using the “irr” package (Gamer et al., 2019). Trial-level agreement was near perfect as indicated by a two-way absolute-agreement single-measure intraclass correlation (ICC = .989, 95% CI [.988, .990]). *N* = 3 participants were excluded from the startle analysis because they showed nonresponses or missings to >66% of the startle probes, including the initial startle habituation phase. The single trial startle responses were entered into the analyses without aggregation (participant’s individual trials *M* = 41.90, *Mdn* = 42, *SD* = 4.40) and without standardization, due to multilevel modelling using random effects to account for interindividual differences in absolute startle magnitude. Internal consistency of the startle responses was estimated using Cronbach’s alpha across single trials separately for each cue within each condition and ranged from α = .892 to α = .975 (N interstimulus interval: α = .918; N countdown: α = .896; P interstimulus interval: α = .892; P countdown: α = .975; U interstimulus interval: α = .914; U countdown: α = .894).

### Electroencephalogram recording and processing

EEG data were acquired via 61 Ag/AgCl electrodes mounted on a cap with equidistant electrode sites (Easycap, Herrsching, Germany) and two 32-channel BrainAmp amplifiers (Brain Products GmbH, Gilching, Germany). EEG signals were recorded with a band-pass filter of 0.01 to 250 Hz and digitized continuously at a sampling rate of 1000 Hz. The recording reference was located between AF3 and Fz, the ground electrode between AF4 and Fz. An external electrode was placed at the left infraorbital site for vertical eye movements. Processing of the EEG data was performed in Brain Vision Analyzer version 2.3 (Brain Products GmbH, Gilching, Germany). The data were band-pass filtered with a low cut-off of 0.1 Hz and a high cut-off of 30 Hz (24 dB/oct roll-off) as well as a notch filtered (50 Hz). To detect and correct for ocular artifacts, an independent component analysis (ICA; Jung et al., 2000) was used, in which relevant components were semi-automatically identified and manually checked by visual inspection of the scalp topography, the component activation, and the inspection of the corrected EEG signal. The continuous data were then re-referenced to the average of all scalp electrodes and segmented into startle probe-locked epochs (−200 ms to 800 ms relative to the startle probe). Segments containing artifacts (i.e., absolute voltage range exceeding 200 μV, voltage step exceeding 50 μV between consecutive data points, or maximum voltage difference of <0.5 μV within 100 ms intervals) were removed. Segments were then baseline corrected (i.e., −200 ms to 0 ms relative to the startle probe). No participant was excluded based on the predefined exclusion criterion (i.e., >50% of segments at any cue (i.e., interstimulus interval or countdown) in any condition (i.e., N, P, or U) after artifact rejection). The quantification of ERPs was based on visual inspection of the grand-averaged waveforms and topographical distributions: The probe N1 (scored at FCz) was quantified as the ± 25 ms area around the most negative peak (approx. between 60 ms and 170 ms). The probe P3 (scored at Pz) was quantified as mean activity 250 - 350 ms (Polich, 2007). To determine split-half reliability, we calculated Spearman-Brown corrected correlations of odd and even trials separately for the conditions (i.e., N, P, U). For N1, these correlations were high across conditions, with *r* = .92 in the N and *r* = .88 in both the P and U conditions. For P3, correlations were *r* = .79 in the N condition, *r* = .83 in the P condition, and *r* = .84 in the U condition.

### Skin conductance recording and processing

SCRs were measured using two Ag/AgCl electrodes with 12 mm sensor diameter placed at the distal and proximal hypothenar eminence of the non-dominant hand. Electrodes were applied five minutes prior to measurement onset. A low constant-voltage of 0.5 V was passed through the electrodes. Preprocessing of SCR involved downsampling to 10 Hz, a continuous decomposition analysis using Ledalab (Benedek & Kaernbach, 2010) running in MATLAB R2025b (MathWorks®, Natick, Massachusetts, USA). The data were optimized using the “optimize” function implemented in Ledalab. SCRs were automatically scored in a response onset window of 0.9 s to 3.5 s after stimulus onset. For this, the amplitudes were required to exceed a minimum amplitude threshold of 0.01 µS. SCRs were scored in response to the interstimulus intervals in conditions N and U and the “5” of the countdown in conditions N, P, and U, in trials without a preceding electrotactile stimulation in the time window of 2.6 s before the onset of the countdown. Participants with no discernible responses to >66% of aversive stimuli were excluded from SCR analyses (*n* = 13).

### Ratings

Participants were asked to rate how anxious they were at the presentation of each cue (i.e., interstimulus interval and countdown) in each condition (i.e., N, P, and U) on a 7-point Likert scale from “not at all anxious” to “extremely anxious”.

### Statistical analyses

The analyses were performed in R version 4.5.2 and RStudio version 2026.1.1.403. Multilevel models were estimated using the packages “lme4” (Bates et al., 2015), the package “emmeans” (Lenth et al., 2021) and “lmerTest” (Kuznetsova et al., 2017). Separate models were fitted for each outcome: startle, N1, P3, SCR, and ratings. Following the five general exclusions, the analytic sample comprised 155 participants for startle, 158 for N1 and P3, 145 for SCR, and 158 for ratings. Trial-level data were analyzed for startle and SCR, whereas EEG data for the N1 and P3 were aggregated per cue and condition. Physiological responses were nested within participants. Effect sizes for fixed effects were quantified using semi-partial *R*^2^, calculated with the “r2glmm” package (Jaeger, 2017). Continuous questionnaire predictors were grand-mean centered. Confirmatory models included condition, diagnostic group, and their interaction:

*Y ∼ condition × group + (1 + condition | participant)*

To test dimensional predictors, we fitted models including intolerance of uncertainty, anxious apprehension, anxious arousal, and depressive symptoms, operationalized by the IUS, PSWQ, MASQ, and BDI-II:

*Y ∼ condition × IUS + condition × PSWQ + condition × MASQ + condition × BDI + (1 + condition | participant)*

To compare categorical and dimensional predictors, significant dimensional predictors were subsequently entered together with diagnostic group in combined models. Where preregistered, combined models were simplified by backward elimination of fixed effects, beginning with the highest-order interactions while retaining lower-order terms involved in significant interactions. The preregistered model reduction was used to simplify the combined categorical-dimensional models by removing unsupported higher-order interactions. This was intended to reduce model complexity given the overlap among the individual-difference measures. Random-effects structures included random intercepts and condition slopes by participant whenever supported by model convergence. In case of non-convergence, the random-effects structure was simplified. Fixed effects were evaluated using Satterthwaite-approximated degrees of freedom. Significant interactions involving categorical predictors were followed up with Tukey-corrected pairwise comparisons of estimated marginal means. Interactions involving continuous predictors were followed up with comparisons of estimated slopes across factor levels. The significance threshold was set to α = .05.

## Results

See Supplement A for descriptive statistics of all outcomes (i.e., ratings, startle, SCR, N1, and P3).

### Ratings

The final model for ratings after the preregistered model selection was

*Rating ∼ MASQ + IUS + condition × group × BDI + (1 + condition | participant)*

The analysis of the anxiety ratings yielded significant main effects of condition, BDI, IUS, and MASQ (for the full model results see Supplement B). Participants rated the conditions as anxiety inducing U > P > N (Supplement B). Overall, higher IUS scores were associated with higher anxiety ratings in all conditions (*b* = 0.03, *SE* = 0.01, *t*(921) = 3.07, *p* = .002, *R*^2^ = .044). Similarly higher MASQ scores were associated with higher anxiety ratings in all conditions (*b* = 0.03, *SE* = 0.01, *t*(921) = 2.08, *p* = .038, *R*^2^ = .021). Further, a condition × group × BDI interaction emerged (*F*(6, 151) = 2.49, *p* = .025, *R*^2^ = .028). Follow-up analyses indicated that in the predictable threat condition, BDI scores were negatively associated with ratings in the control group (*b* = −0.086, *SE* = 0.037, 95% CI [−0.159, −0.014], *p* = .020), whereas the corresponding slopes were not significant in the OCD, PHOB, or SAD groups. However, Tukey-adjusted contrasts of BDI slopes between groups did not reach the significance threshold. Together, this indicates that intolerance of uncertainty and anxious arousal were associated with increased ratings across all conditions and groups.

### Skin conductance responses

The final model for SCR after the preregistered stepwise model reduction was

*SCR ∼ condition × group + condition × MASQ + (1 | participant)*

The model yielded main effects of condition (*F*(2, 5916) = 179.08, *p* < .001, *R*^2^ = .006) and group (*F*(3, 146) = 2.66, *p* = .050, *R*^2^ = .002), which were qualified by a significant condition × group interaction (*F*(6, 5916) = 5.31, *p* < .001, *R*^2^ = .008, see Supplement C). Group contrasts within each condition indicated that groups did not differ in N or P (all *p*s ≥ .346). In U, however, the OCD group showed larger SCRs than controls (*b* = 0.07, *SE* = 0.016, *t* = 4.34, *p* < .001), PHOB (*b* = 0.07, *SE* = 0.016, *t* = 4.38, *p* < .001), and SAD (*b* = 0.05, *SE* = 0.016, *t* = 3.44, *p* = .003), whereas no other group contrasts reached significance.^1^ Thus, the interaction was driven by selectively enhanced SCRs during unpredictable threat anticipation in OCD.

Concerning dimensional effects, the model yielded a condition × MASQ interaction (*F*(2, 5914) = 6.72, *p* = .001, *R*^2^ = .002). Follow-up analyses showed that MASQ was not significantly related to SCRs in the N condition (*b* = 0.00009, *SE* = 0.00069, *t*(194) = 0.13, *p* = .899) and in the P condition (*b* = −0.00078, *SE* = 0.00079, *t*(338) = −0.98, *p* = .329), but was negatively associated with SCRs in the U condition (*b* = −0.00185, *SE* = 0.00071, *t*(215) = −2.61, *p* = .010). Correspondingly, the MASQ slope differed significantly between U and N (Δslope = 0.00193, *SE* = 0.00053, *t*(5913) = 3.67, *p* < .001), whereas the contrasts between N and P were not significant (*p* = .366, P and U, *p* = .234). Thus, higher anxious arousal was associated with reduced SCRs specifically during unpredictable threat anticipation. There was no significant condition × group × MASQ interaction (*F*(6, 5900) = 0.44, *p* = .850).

### Startle

The final model for startle after the preregistered stepwise model reduction was

*Startle ∼ condition × group × IUS + (1 | participant)*.

The pattern of task effects was as expected from previous studies (U > P > N, see Supplement D).

**Fig. 1:**
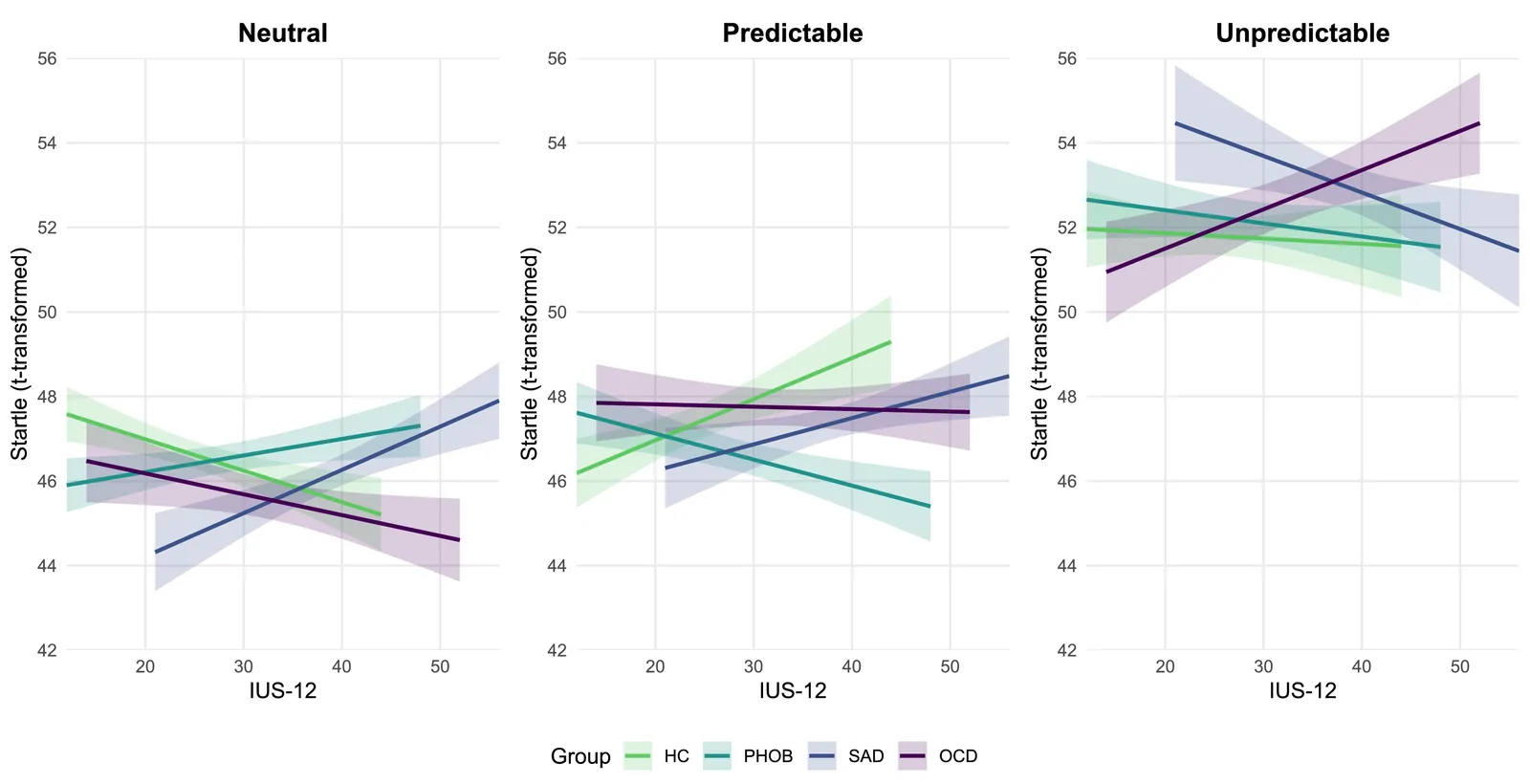
Associations between intolerance of uncertainty and startle responses.

*Note.* Associations between intolerance of uncertainty (IUS-12) and startle magnitude across experimental conditions. Groups include unaffected controls (HC), specific phobia (PHOB), social anxiety disorder (SAD), and obsessive-compulsive disorder (OCD). Solid lines represent fitted linear trends based on the observed data, shaded bands indicate ±1 standard error.

Concerning individual difference effects, the model yielded a trendwise significant condition × group interaction (*F*(6, 6289) = 2.07, *p* = .053, *R*^2^ = .002) which was further specified by a significant condition × group × IUS interaction (*F*(6, 6289) = 2.60, *p* = .016, *R*^2^ = .003). Tukey-corrected contrasts of the slopes of IUS per level of condition and group led to one significant difference of the slopes in the control group between conditions N and P (Δslope[N–P] = −2.31, *SE* = 0.68, *t*(6289) = −3.42, *p* = .031), indicating that participants from the control group displayed increased startle responses in the P condition compared with the N condition if they had increased IU scores (Fig. 1). All other contrasts did not reach the predefined significance threshold after Tukey correction (*p*s > .459). Following the condition × group interaction with contrast of the estimated marginal means between groups per level of condition, we found that the control group showed increased startle in the P condition compared with participants from the PHOB group (*b* = 90.32, *SE* = 30.50, *t*(154) = 2.96, *p* = .019). However, this effect might not be specific to the P condition, as it was also observable, yet not significant in the U condition (*b* = 78.06, *SE* = 30.50, *t*(153) = 2.56, *p* = .055), and, to a lesser extent, in the N condition (*b* = 65.92, *SE* = 30.50, *t*(154) = 2.16, *p* = .139) and might thus reflect a main effect of group. No further significant contrasts emerged between groups. Overall, startle responses varied by condition, group, and intolerance of uncertainty, with higher intolerance of uncertainty associated with greater P vs. N startle differentiation in the control group, and controls showing generally higher startle responses than the PHOB group.

### N1

The final model for N1 after stepwise model reduction was

*N1 ∼ condition × MASQ + condition × BDI + condition × group × PSWQ + (1 | participant)*

Across participants, N1 showed a U > P = N pattern (see Supplement E).

**Fig. 2:**
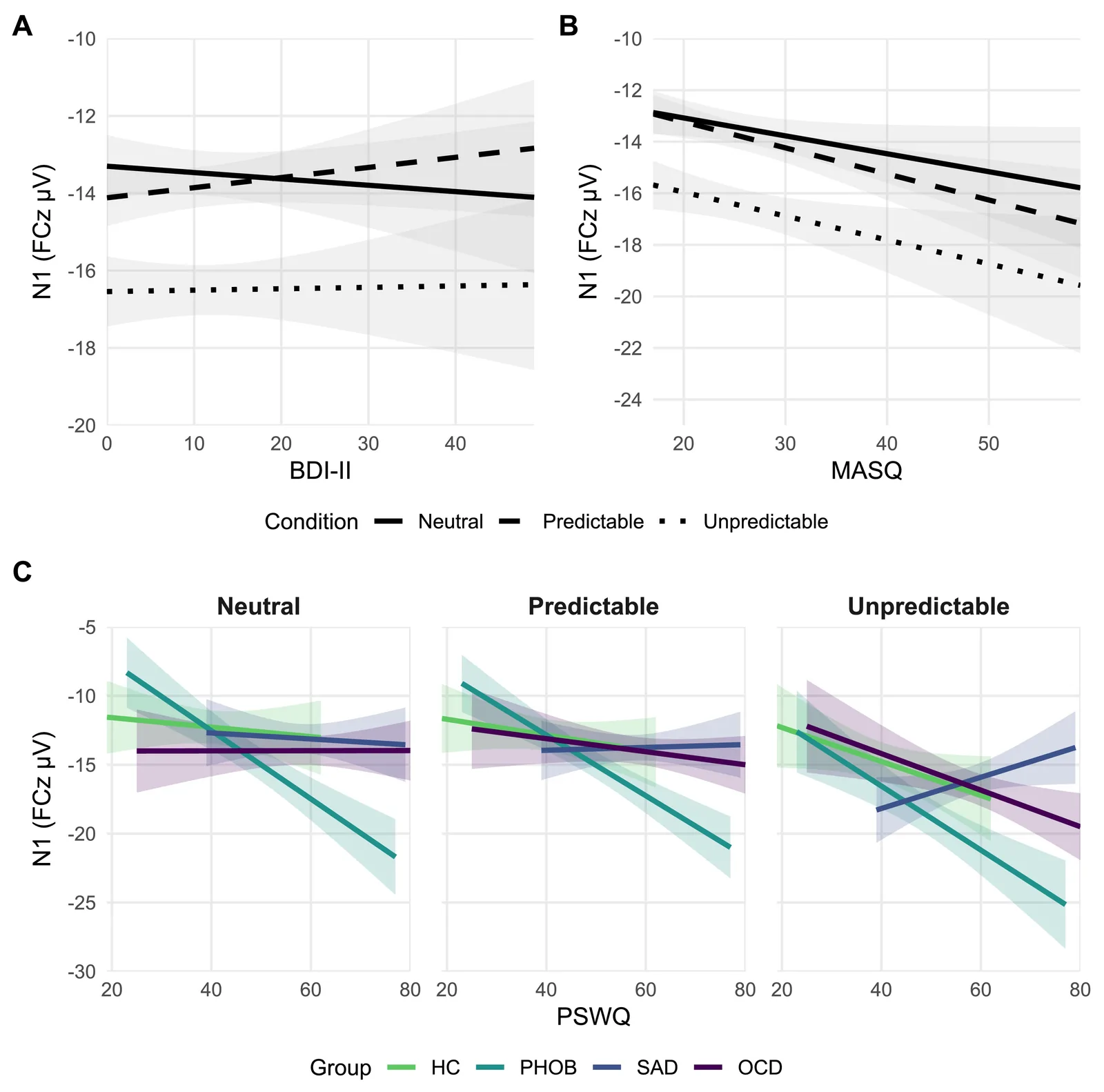
Associations between clinical measures and N1.

*Note*. **A.** Association between Beck Depression Inventory-II (BDI-II) scores and probe-locked N1 amplitude. **B.** Association between Mood and Anxiety Symptom Questionnaire Anxious Arousal subscale (MASQ) scores and probe-locked N1 amplitude. **C.** Association between Penn State Worry Questionnaire (PSWQ) scores and probe-locked N1 shown per group. Groups include unaffected controls (HC), specific phobia (PHOB), social anxiety disorder (SAD), and obsessive-compulsive disorder (OCD). Shaded bands indicate ±1 standard error.

Regarding individual difference effects, a condition × group × PSWQ interaction emerged (*F*(6, 770) = 2.24, *p* = .038, *R*^2^ = .009). Contrasts of the PSWQ slopes within groups yielded a significant difference between the U and N conditions (*b* = 0.17, *SE* = 0.06, *t*(770) = 2.79, *p* = .015), indicating a stronger PSWQ-related increase in N1 in the U vs. N in patients with OCD. Other contrasts did not reach significance after Tukey correction (*p*s > .171). The PHOB group showed a consistently negative PSWQ-N1 association across all three conditions (*b* = −0.25, *SE* = 0.08, *t*(148) = −3.02, *p* = .003), indicating that higher PSWQ scores were associated with a more negative (i.e., increased) N1. Further, a condition × BDI interaction emerged (*F*(2, 770) = 5.04, *p* = .007, *R*^2^ = .007). BDI was positively associated with N1 in the P condition (*b* = 0.19, *SE* = 0.08, *t*(182) = 2.51, *p* = .013), whereas the corresponding slopes were not significant in N (*b* = 0.06, *SE* = 0.08, *t*(182) = 0.79, *p* = .429) or U (*b* = 0.14, *SE* = 0.08, *t*(182) = 1.79, *p* = .075). The BDI slopes differed significantly between P and N (Δslope[N–P] = −0.13, *SE* = 0.04, *t*(770) = −3.16, *p* = .005), while differences between N and U (*p* = .160) and between P and U (*p* = .379) did not reach significance. This pattern of results indicates that higher BDI scores are associated with a reduced N1 during predictable threat anticipation. In addition, a condition × MASQ interaction emerged (*F*(2, 770) = 3.04, *p* = .048, *R*^2^ = .004). This was driven by a significant difference of MASQ slopes between N and P (*b* = 0.13, *SE* = 0.05, *t*(770) = 2.36, *p* = .049), whereas differences between N and U (*p* = .849) and P and U (*p* = .167) did not reach significance. Overall, N1 was enhanced during unpredictable threat anticipation, with modulation by individual differences. Worry was associated with stronger unpredictable N1 responses in patients with OCD and across conditions in the PHOB group, depressive symptoms were associated with blunted N1 during predictable threat, and anxious arousal was associated with increased N1 during predictable threat relative to safety (Fig. 2).

### P3

The final model for the probe-locked P3 after preregistered stepwise model reduction was

*P3 ∼ condition + MASQ + (1 + condition | participant)*

Generally, the P3 was suppressed in threat conditions (U = P < N, see Supplement F). Concerning individual differences, the model further yielded a main effect of MASQ (*F*(1, 156) = 9.48, *p* = .002, *R*^2^ = .106). Higher anxious arousal was associated with lower P3 amplitudes across conditions (*b* = −0.12, *SE* = 0.04, *t*(156) = −2.71, *p* = .008). The condition × MASQ interaction was not significant (*F*(2, 156) = 0.16, *p* = .854).

### Exploratory latent profile analysis

Latent profile analysis was conducted as an exploratory person-centered analysis to identify participants with similar multimodal response patterns in threat anticipation and to integrate results across outcomes. The latent profile analysis was based on z-standardized, condition-averaged physiological indicators (startle, SCR, N1, P3) across the three conditions (N, P, U) in the complete-case sample without missing data in any outcome (*N* = 143). One to five profile Gaussian mixture models with freely estimated profile means, equal indicator variances, and zero within-profile covariances were compared using information criteria, entropy, minimum class size, posterior classification probabilities, and substantive interpretability. Fit improved markedly from one to three profile solution with significant BLRTs supporting the addition of profiles up to three classes (Supplement G). Although four and five profile solutions yielded marginal additional improvement in BIC, they produced smaller and less clearly differentiated profiles. The three-profile solution was therefore retained as the most parsimonious and interpretable solution with adequate classification quality (BIC = 4802.11, entropy = .85, minimum class assignment probability = .91).

**Fig. 3:**
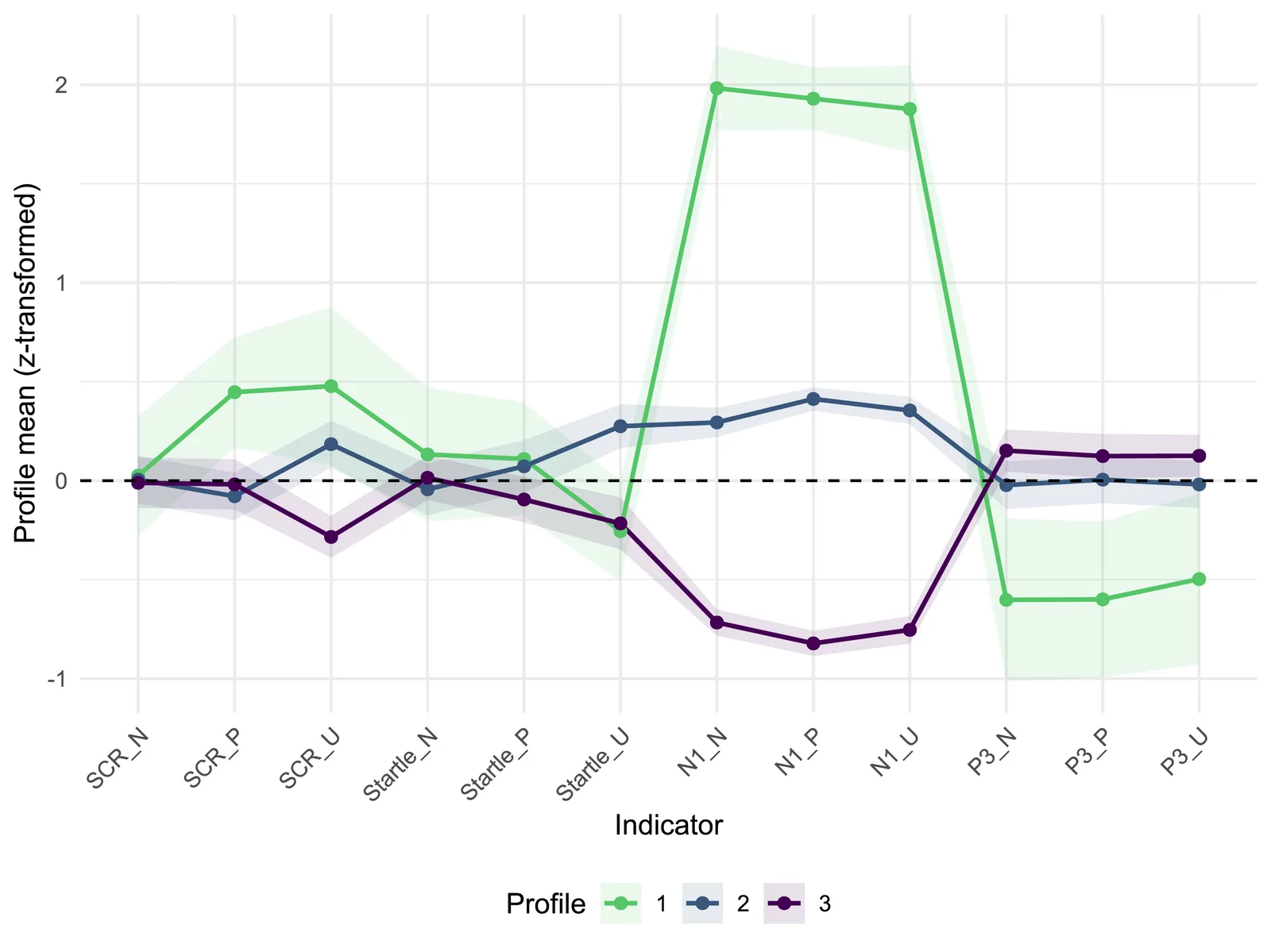
Profiles retained from latent profile analysis.

*Note.* Shaded error bars represent ± 1 standard error. For visualization, N1 and P3 were recoded such that higher *z*-values (i.e., *z* > 0) represent increased sensitivity to threat. Conditions include neutral (N), predictable threat (P), and unpredictable threat (U).

Retained solutions were interpreted based on their multisystem response patterns, and modal profile membership was then merged back into the full dataset to examine external validity in relation to diagnostic group and clinical variables using omnibus ANOVAs and chi-square/Fisher tests, followed by corrected post hoc comparisons. As such, the retained profiles differed more clearly in their multisystem response configuration than in purely condition-specific response to unpredictable threat (Fig. 3, Figure S2 in Supplement H). Profile 1 (*n* = 14; 9.79% of the sample) was characterized by pronounced early sensory processing (N1) across all conditions. Profile 2 (*n* = 64; 44.76%) appeared to capture a defensive/autonomic unpredictable threat-reactive response style with increased N1, average P3 suppression, and both SCR and startle increases to unpredictable threat. Profile 3 (*n* = 65; 45.45%) was characterized by comparatively attenuated neural and peripheral responding, including less negative N1, indicating less automatic early sensory processing of stimuli regardless of condition, slightly lower P3, and both lower startle and SCR during unpredictable threat compared with Profile 2. Profile 3 suggests generalized physiological dampening particularly under unpredictable threat, consistent with relatively hyporeactive defensive responding.

To clarify whether the retained physiological profiles reflected general response style or differential sensitivity to the NPU conditions, modal profile membership from the retained three-profile solution was projected back onto the original data and separate mixed models were fit for SCR, startle, N1, and P3. This back-projection was used to determine in which physiological systems the retained profiles actually differed across the N, P, and U conditions (Supplement H). Taken together, the back-projected models indicated that the retained latent profiles are better characterized as broad multisystem response styles rather than distinct forms of condition-specific NPU sensitivity. Only SCR showed a clear profile × condition interaction, suggesting that autonomic responding differentiated the profiles in their sensitivity to threat anticipation, especially under unpredictable threat. In contrast, N1 showed strong profile differences in overall level with only trend-level evidence for differential NPU modulation, while P3 and startle showed largely parallel condition effects across profiles (Supplement H, Figure S2).

### Clinical correlates of latent profiles

To examine the clinical relevance of the physiological profiles, modal profile membership was related to clinical status and diagnostic group composition. Clinical status differed descriptively across profiles (Supplement I, Figure S3), with the highest proportion of clinically affected participants in Profile 2 (56/64; 87.50%) compared with Profile 1 (10/14; 71.43%) and Profile 3 (46/65; 70.77%). The omnibus association was not significant using Pearson’s χ² test (χ²(2, *N* = 143) = 5.75, *p* = .056, Cramér’s *V* = .20), but reached significance using Fisher’s exact test (*p* = .049). Pairwise comparisons suggested that this pattern was mainly driven by a higher clinical proportion in Profile 2 compared to Profile 3, although this contrast did not survive Holm correction (raw *p* = .034, Holm-adjusted *p* = .102). Accordingly, profile membership showed only modest discrimination of clinical status in a logistic ROC analysis (AUC = .622, 95% CI [.524, .719]; Supplement I, Figure S4).

**Fig. 4:**
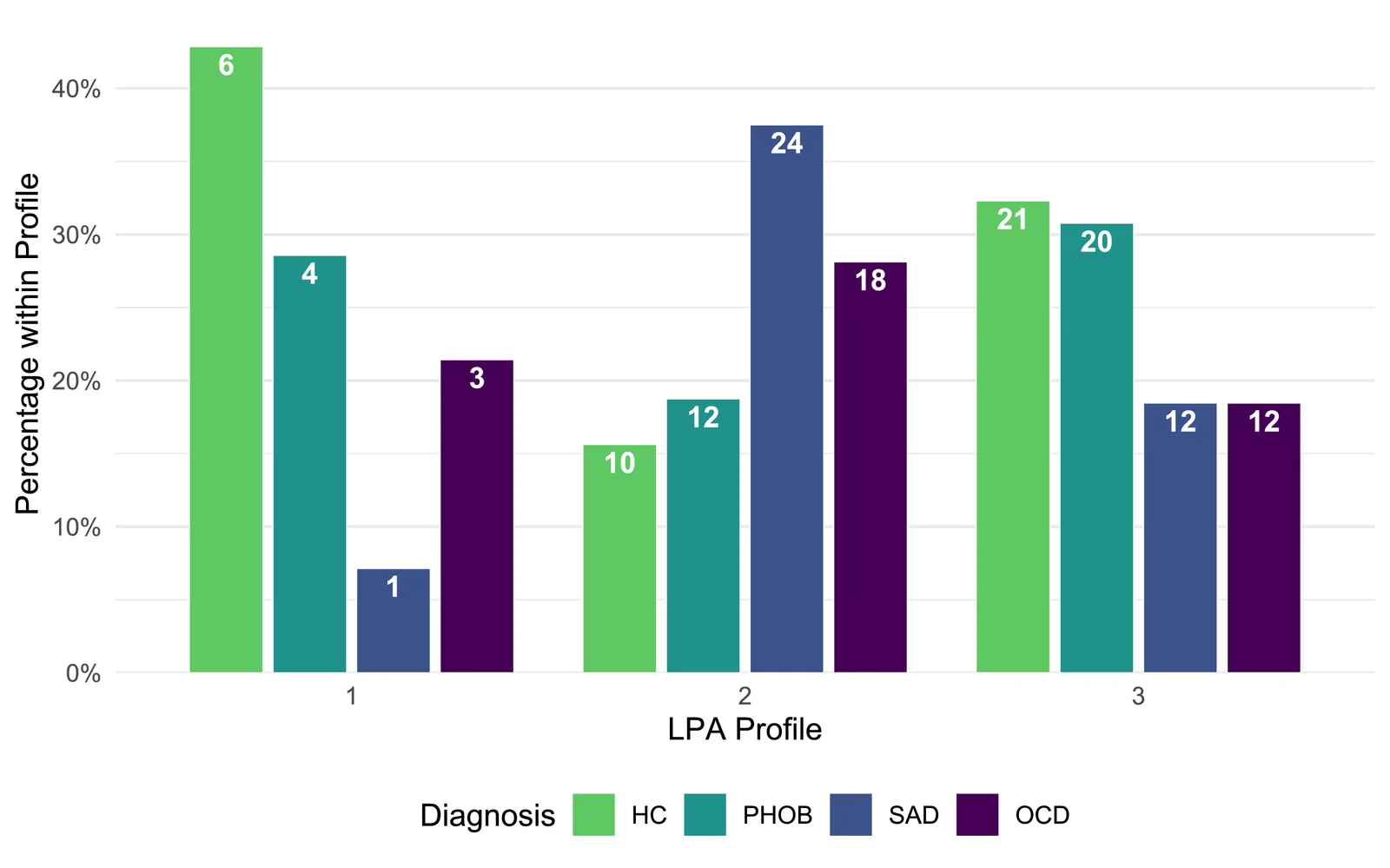
Clinical composition of latent profiles.

*Note.* LPA = latent profile analysis; HC = unaffected controls; PHOB = specific phobia; SAD = social anxiety disorder; OCD = obsessive-compulsive disorder. White digits show the count of participants within each profile.

Notably, diagnostic composition differed significantly across profiles (χ²(6, *N* = 143) = 15.07, *p* = .020, Fisher’s exact *p* = .016, Cramér’s *V* = .23). Descriptively, Profile 2 contained the largest proportion of patients with SAD and OCD whereas controls were comparatively underrepresented. In contrast, Profile 3 contained relatively larger proportions of unaffected controls and patients with specific phobia, consistent with a less clinically burdened or more circumscribed fear composition. Profile 1 was small and should be interpreted cautiously, but was composed primarily of unaffected controls and patients with specific phobia, with few patients with SAD (Fig. 4). Cellwise residuals indicated that SAD participants were overrepresented in Profile 2 and underrepresented in Profile 3 descriptively, although these cellwise effects did not clearly survive correction for multiple comparisons (Supplement I, Table S9).

**Fig. 5:**
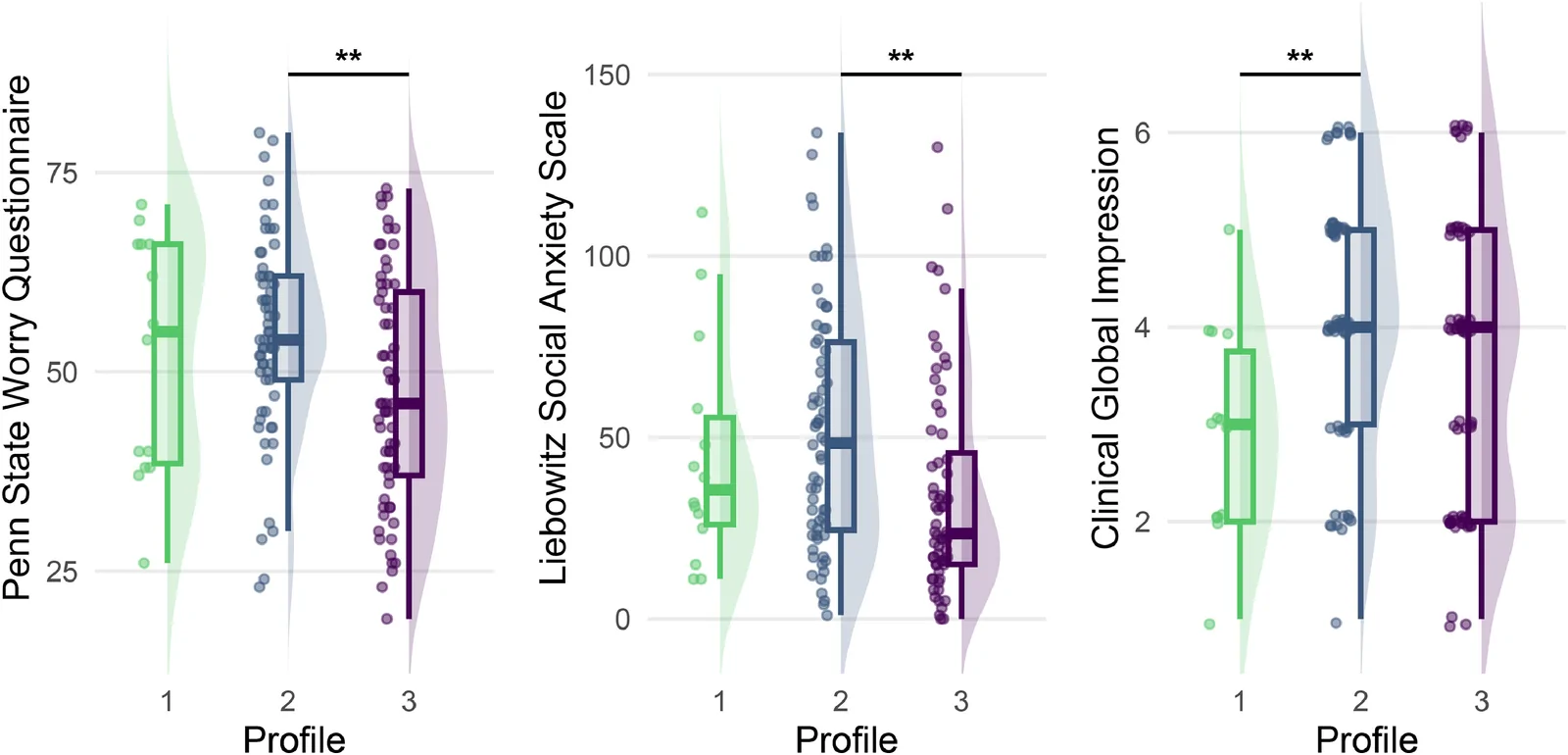
Clinical correlates of the physiological latent profiles.

*Note*. Profile 1 (*n* = 14) contains a smaller sample size than Profiles 2 (*n* = 64) and 3 (*n* = 65). ** = *p* < .01

The group differences of the physiological profiles were further underscored by dimensional differences emerging for worry, social anxiety symptoms and clinician-rated severity (Fig. 5). Specifically, PSWQ differed across profiles (*F*(2, 140) = 4.48, *p* = .013) with higher PSWQ scores in Profile 2 than in Profile 3 (*b* = 7.10, *SE* = 2.38, *t*(140) = 2.98, *p* = .010) whereas Profile 1 did not differ in PSWQ from either profile (*p*s > .479). A similar pattern emerged for social anxiety symptoms (*F*(2, 139) = 4.85, *p* = .009), with Profile 2 showing higher LSAS scores than Profile 3 (*b* = 17.17, *SE* = 9.23, *t*(139) = 3.11, *p* = .006) and Profile 1 not differing significantly from Profiles 2 and 3 (*p*s > .504). Clinician-rated severity (i.e., CGI) also differed across profiles, *F*(2, 138) = 5.29, *p* = .006, with Profile 2 showing greater severity than Profile 1 (*b* = 1.17, *SE* = 0.40, *t*(138) = 2.93, *p* = .011). The difference in CGI between Profiles 2 and 3 was in the same direction but did not survive correction (*b* = 0.53, *SE* = 0.24, *t*(138) = 2.23, *p* = .070), while no significant difference emerged between Profiles 1 and 3 (*p* = .251).

## Discussion

The present study primarily examined how physiological reactivity and sensitivity to unpredictable threat across multiple response systems map onto categorical and dimensional variation across the anxiety and obsessive-compulsive spectrum. The confirmatory analyses provided only limited support for such a systematic mapping. Diagnostic categories did not map onto threat-anticipation physiology in the linear manner predicted by the circumscribed fear-anxious misery framework, and sensitivity to unpredictable threat did not increase uniformly from controls to specific phobia, SAD, and OCD. Instead, diagnostic and dimensional effects were response-system specific. The clearest group-based effect emerged for SCR, in which patients with OCD showed enhanced autonomic responding during unpredictable threat anticipation. Dimensional predictors explained additional variance, but in partly distinct ways across physiological systems: anxious arousal was associated with reduced SCRs during unpredictable threat and lower P3 amplitudes across conditions, intolerance of uncertainty was associated with heightened startle to predictable threat in controls, and worry/anxious apprehension was related to stronger N1 modulation during unpredictable threat anticipation in patients with OCD. As an exploratory extension integrating responses across physiological systems using participants with complete case data across all four physiological outcomes, latent profile analysis identified three multimodal response profiles that cut partly across diagnostic categories. These profiles reflected broad multisystem response styles rather than purely condition-specific NPU sensitivity, with one threat-reactive and clinically enriched profile showing greater diagnostic burden, higher representation of SAD and OCD, elevated worry and social anxiety symptoms, and greater clinician-rated severity. Together, these findings suggest that threat anticipation in anxiety-related psychopathology is best understood as a multisystem and partly transdiagnostic process rather than as a single marker of diagnostic status or unpredictable-threat sensitivity.

At the categorical level, neither preregistered hypothesis received consistent support across physiological systems. The diagnostic categories did not clearly map onto threat-anticipation physiology in the linear manner expectable by the circumscribed fear-anxious misery framework (Lang et al., 2016). We expected that general physiological reactivity would decrease with more generalized anxiety psychopathology. Evidence for this would have been conveyed by main effects of group. Consistent with this hypothesis in a limited sense, unaffected controls showed higher startle responses than patients with specific phobia. However, this effect did not follow the expected clinical severity gradient, as the PHOB group was psychopathologically less affected than the SAD and OCD groups across dimensional measures. Apart from this finding, no support for physiological hyporeactivity emerged in group-based analyses across SCR, N1, and P3. At the dimensional level, some findings were compatible with physiological blunting in participants with higher symptom burden, although this pattern did not converge across outcomes. Specifically, increased BDI-II scores were associated with decreased startle probe-locked N1 in predictable threat anticipation, which could be taken to suggest that early attention to predictable threatening contexts might be decreased as a function of depressive symptoms. In contrast to a prior study (Carsten et al., 2023), higher MASQ scores were associated with greater P3 suppression across experimental conditions, which may reflect increased allocation of later attentional resources to threat-relevant stimuli in participants with higher anxious arousal (MacNamara & Barley, 2018; Nelson et al., 2015). Simultaneously, increased anxious arousal was also associated with decreased SCR, especially in unpredictable threat anticipation, indicating less sympathetic activation. Thus, self-reported anxious arousal showed divergent associations across physiological systems, with greater P3 suppression but reduced autonomic responding at the level of SCR. These associations may reflect partly distinct components of threat processing rather than a contradictory pattern. Greater P3 suppression could indicate stronger attentional engagement with the threat context, whereas lower SCR may reflect reduced peripheral sympathetic activation, potentially indexing differences in attentional engagement versus autonomic regulation.

At the categorical level, evidence for the predicted increase in sensitivity to unpredictable threat with proximity to the “anxious misery” pole was limited. Patients with OCD showed enhanced SCRs specifically during unpredictable threat anticipation. Extending these findings at the level of early sensory processing, PSWQ was associated with stronger N1 during unpredictable threat anticipation in OCD patients. Group-based differences for unpredictable threat anticipation emerged specifically in patients with OCD, which raises the question of whether this pattern of results represents an effect of broad anxiety-related psychopathology or symptoms more specific to OCD. Although anxiety disorders and OCD share symptomatic overlap, are highly comorbid (Cheng et al., 2025), and are commonly conceptualized within the broader internalizing spectrum (Kotov et al., 2017, 2022), they differ with respect to cognitive aspects and symptom expression (American Psychiatric Association, 2013; Goodwin, 2015). As such, OCD might not represent the stereotypical expression of “anxious misery” (Lang et al., 2016). Future studies should therefore include patients with GAD to disentangle whether the observed effects are more closely linked to generalized anxiety-related psychopathology or specific to OCD, for which the present findings provide tentative evidence.

Taken together, the confirmatory analyses provided only limited support for the preregistered hypotheses. Although several clinical and dimensional associations reached statistical significance, most interaction effects were small (semi-partial *R*^2^ < .01), indicating that they accounted for only a limited proportion of outcome variance. Neither diagnostic categories nor individual symptom dimensions fully captured the heterogeneity of physiological responding. This raises the possibility that clinically meaningful variation may be organized less by diagnostic group membership alone but rather by broader configurations of physiological responses across systems. To address this possibility, we used exploratory latent profile analysis to examine whether multimodal physiological indicators formed distinct response profiles in a data driven analysis and related those to diagnoses and symptom dimensions.

In the latent profile analysis, three profiles emerged that differed primarily in their overall level of physiological responding across systems. To determine whether the retained physiological profiles captured general patterns of responding or differential modulation by NPU condition, profile membership was projected back onto the original data. This back-projection revealed main effects of condition across all outcomes, main effects of profile across outcomes except startle, and a profile × condition interaction only for SCR. Thus, the exploratory, data-driven profiles appeared to distinguish participants primarily by general response levels across physiological systems, rather than by differential sensitivity to unpredictable threat per se. However, for SCR (and descriptively for N1) profile-specific threat anticipation response patterns were evident. Profile 2 showed increased SCR to unpredictable threat and somewhat more graded threat-related potentiation of N1 amplitudes than Profile 3. At the same time, Profile 2 included more participants with a diagnosis (87.50%) than Profile 3 (70.77%) and Profile 1 (71.43%), which was further underscored by a larger proportion of patients with SAD and OCD in Profile 2, as well as higher PSWQ scores and LSAS scores and clinician-rated severity. Thus, Profile 2, which encompassed nearly half of the sample, was both more threat-reactive and descriptively enriched for clinically affected participants. This may suggest that a moderately threat-reactive multimodal physiological response pattern, as evident in Profile 2, characterized the more severely affected, worrying, and socially anxious patients in the sample. In contrast, the low-reactivity profile (i.e., Profile 3), despite comprising a similarly sized group, was associated with descriptively lower clinician-rated severity and comparably lower SCR in unpredictable threat anticipation, a pattern mirrored descriptively in startle responses, as well as an attenuated early sensory processing (N1). Together, these data-driven profiles do not lend support to the idea of a general physiological blunting with increasing severity (Lang et al., 2016; McTeague & Lang, 2012), as has empirically been shown for survivors of noninterpersonal trauma (Kreutzer & Gorka, 2021; Lieberman et al., 2020). Rather, these results suggest that combining multimodal physiological indices during the NPU-threat test may capture variation that is not readily apparent when considering outcomes individually. Specifically, Profile 2 was characterized by stronger sympathetic responding and early sensory processing during unpredictable threat, as indicated by stronger SCR increases and more pronounced N1 threat modulation relative to Profile 3. If replicated, this pattern may be relevant to models proposing that uncertain contexts are associated with heightened threat salience and physiological responding (Brosschot et al., 2017, 2018; Van den Bergh et al., 2021). This interpretation aligns with the uncertainty and anticipation model of anxiety, in which uncertain future threat is thought to promote maladaptive anticipatory responding through heightened threat expectancies, biased threat attention, reduced use of safety information, and exaggerated physiological reactivity to uncertainty (Grupe & Nitschke, 2013). At the same time, the association of Profile 2 with SAD/OCD and greater clinical severity provides an exploratory indication that clinical burden may be associated with multisystem patterns of threat-context engagement rather than generalized physiological blunting (Lang et al., 2016; McTeague & Lang, 2012).

As shown earlier, clinical effects in the NPU-threat test have been heterogeneous across studies (Gorka et al., 2014; Grillon et al., 2017; Hoge et al., 2024; Katz et al., 2018; Stevens et al., 2018). Through the lens of these prior heterogeneous findings, the present results provide three insights and avenues for future research. First, the present study provides direct comparisons of threat anticipation between disorder categories. Previous NPU studies with two or more clinical groups have usually not shown uniform increases in unpredictable threat anticipation in startle responses (Gorka et al., 2017; Grillon et al., 2017; Katz et al., 2018; Shankman et al., 2013). Rather, the direct comparisons between disorder categories allowed for more nuanced differentiation of diagnostic groups. The present findings provide data for direct disorder comparisons across startle, SCR, N1, and P3, which can facilitate meta-scientific endeavors, especially network meta-analysis. This is highly needed, because there is no meta-analytic effect integration across NPU-threat test studies available yet. Second, the present study adds to the limited knowledge on how dimensional conceptualizations of psychopathology can provide incremental insight into the clinical correlates of physiological responses during unpredictable threat anticipation, an approach that is closely aligned with the Research Domain Criteria framework (Cuthbert, 2015, 2020). While subclinical studies often include associations with questionnaire data (e.g., Carsten et al., 2022, 2023; Morriss et al., 2020; Nelson et al., 2016; Nelson et al., 2015; Papenfuss et al., 2021; Rutherford et al., 2020), reports of dimensional effects in clinical studies are scarce. To connect and integrate subclinical findings and estimate their generalizability to clinical samples, questionnaire-physiology associations in clinical samples are necessary, which the present study provides. Third, the same strategies that help address clinical heterogeneity can also increase analytic complexity. Specifically, including direct disorder comparisons, broadening the spectrum of physiological outcomes, and adding dimensional effects can complicate interpretability of the results. Thus, data-driven approaches may provide a useful exploratory strategy for integrating multivariate response patterns, as illustrated by the present exploratory latent profile analysis. Importantly, the level of complexity could be reduced at each of the factors that produce complexity. As such, dimensional reductions such as factor analysis on questionnaire data would also be feasible. Together, these points suggest that progress in clinical NPU research may depend less on identifying a single robust marker of unpredictable-threat sensitivity and more on systematically characterizing how diagnostic status, symptom dimensions, and physiological systems jointly structure anticipatory responding to impending threat. At the same time, data-driven approaches that integrate response patterns across outcomes can reduce complexity but, as a cost, might limit generalizability and provide insights that are specific to the sample under study.

### Limitations

The exploratory latent profile analysis should be interpreted with caution. Although the retained profiles showed clinically meaningful correlates, the external validity of physiological response levels and thus their generalizability remain to be tested. In particular, the clinically enriched threat-reactive Profile 2 warrants validation in an independent sample. Because the profiles were based on *z*-standardized physiological indicators, they describe relative response configurations within the present sample rather than absolute physiological thresholds. Accordingly, the resulting profiles are dependent on the distribution and reliability of the physiological indicators in this specific sample. In addition, the latent profile analysis was based on complete cases and downstream analyses relied on modal profile assignment, which does not fully account for classification uncertainty. Moreover, Profile 1 contained relatively few participants (*n* = 14), and no external or cross-validation of the profile solution was performed. Thus, the latent profile analysis should be regarded as an exploratory, hypothesis-generating extension of the preregistered confirmatory analyses rather than as evidence for established clinical subtypes. A related limitation concerns the clinical composition of the sample. Because participants in the clinical groups frequently had comorbid disorders, group differences cannot be attributed exclusively to the target diagnosis (Table 1; Supplement J), although the dimensional analyses captured broader transdiagnostic symptom variation. The groups examined here did not cover the full anxiety disorder spectrum, which may have reduced the ability to detect the expected gradient from circumscribed fear to anxious misery (Lang et al., 2016; McTeague & Lang, 2012). In particular, future studies should include patients with GAD, panic disorder, agoraphobia, and separation anxiety to determine whether the observed effects are linked to broad anxiety-related psychopathology or to more disorder-specific processes. A further, partly related limitation concerns psychotropic medication use, which differed across groups, with the highest proportion observed in the OCD group. Sensitivity analyses indicated that the OCD-specific increase in SCR during unpredictable threat was retained both in unmedicated participants and after statistically accounting for medication use. Thus, medication use is unlikely to fully explain the primary SCR group effect, although medication-related influences on physiological responding more generally cannot be ruled out. At the same time, excluding medicated patients would have reduced the clinical representativeness of the sample. Further, the preregistered analytical strategy used a backward elimination procedure to simplify combined categorical-dimensional models. Although this reduced model complexity, data-dependent variable selection can compromise model stability, coefficient estimates, and the validity of *p*-values and confidence intervals (Heinze et al., 2018). Thus, although the selection procedure was specified a priori, the resulting model structures remain data-dependent and may be sensitive to the specific sample. Relatedly, given the number of outcome-specific models and interaction tests, greater weight should be placed on the overall pattern of findings than on isolated dimensional associations, particularly those emerging from higher-order interactions. At the same time, convergence across physiological outcomes was not necessarily expected, as the measures index partly distinct components of threat responding. These measures were chosen for their complementary properties, enabling assessment of different components of threat processing under uncertainty.

Finally, the definition of the control group may have affected the size and interpretation of clinical contrasts. The controls were screened to exclude the target disorders (i.e., specific phobia, SAD, and OCD) but were not necessarily free of any psychopathology. This increases the ecological validity of the control group and may better approximate the general population, in which lifetime psychopathology is common (Caspi et al., 2020). At the same time, it may have reduced differences between controls and clinical groups, thereby increasing the likelihood of false-negative findings for effects of general psychopathology. Consequently, the present design may have been relatively conservative for detecting broad clinical effects, while being more informative for identifying effects specific to the disorders under study.

### Conclusion

In conclusion, the present findings provide only limited support for the idea that physiological threat anticipation follows a simple gradient from circumscribed fear to anxious misery. Instead, effects differed across physiological systems, with the clearest group difference emerging for SCRs during unpredictable threat anticipation in OCD. The exploratory latent profile analysis further suggested that broader multisystem response patterns may capture clinically meaningful differences that are not fully explained by diagnostic categories alone. Within the present sample, a threat-reactive profile emerged that was characterized by stronger autonomic and early sensory engagement particularly during unpredictable threat, a higher proportion of patients with SAD and OCD, and elevated worry, social anxiety symptoms, and clinician-rated severity. Overall, threat anticipation in anxiety-related psychopathology appears to be better understood as a multimodal and partly transdiagnostic process than as a single physiological marker of diagnostic status or unpredictable-threat sensitivity.

## Funding

This research was supported by the German Research Foundation, grant awarded to Anja Riesel DFG-Grant RI-2853/2-1).

## CRediT author statement

**Hannes Per Carsten:** Conceptualization, Methodology, Software, Validation, Formal analysis, Investigation, Data Curation, Writing – Original Draft, Writing – Review & Editing, Visualization, Project administration. **Kai Härpfer:** Conceptualization, Methodology, Validation, Investigation, Data Curation, Writing – Review & Editing, Project administration. **Franziska Magdalena Kausche:** Conceptualization, Methodology, Validation, Investigation, Data Curation, Writing – Review & Editing, Project administration. **Anja Riesel:** Conceptualization, Methodology, Software, Validation, Resources, Writing – Review and Editing, Supervision, Project administration, Funding acquisition.

## Declaration of generative AI and AI-assisted technologies in the manuscript preparation process

ChatGPT (OpenAI, Inc.), version GPT 5, was used for language editing purposes and the improvement of readability. After using this tool, the authors reviewed and edited the content as needed and take full responsibility for the content of the published article.

## Declaration of competing interest

The authors declare that they have no known competing financial interests or personal relationships that could have appeared to influence the work reported in this paper.

## Footnotes

1 Sensitivity analyses indicated that the SCR condition × group effect was retained both when analyses were restricted to unmedicated participants (*n* = 130, *F*(6, 5305) = 5.17, *p* < .001) and when medication status and its interaction with condition were included in the model (*F*(6, 5912) = 5.70, *p* < .001). In both analyses, the OCD group continued to show higher SCRs during unpredictable threat than the other three groups (all Tukey-adjusted *p*s ≤ .007).
