# Supplementary material for: Transdiagnostic physiological correlates of unpredictable threat anticipation
Source: Int J Clin Health Psychol. 2026 Sep 14;26(3):100723. doi: 10.1016/j.ijchp.2026.100723 (PMC13594725; doi:10.1016/j.ijchp.2026.100723)
Supplement: Supplementary file 1 [file mmc1.pdf]

Supplementary Material to

Transdiagnostic Physiological Correlates of Unpredictable Threat Anticipation

Table of Contents:

Supplement A: Descriptive statistics 2

Supplement B: Multilevel model for ratings 3

Supplement C: Multilevel model for skin conductance responses 5

Supplement D: Multilevel model for startle 7

Supplement E: Multilevel model for N1 9

Supplement F: Multilevel model for P3 11

Supplement G: Latent profile analysis model indices 12

Supplement H: Physiological differences in latent profiles 14

Supplement I: Clinical correlates of latent profiles 17

Supplement J: Prevalence of lifetime comorbid depressive disorders 20

Supplement A: Descriptive statistics

Table S1

Outcome	Condition, Cue	**Full Sample** *M* (*SD*)	**Control**	**PHOB**	**SAD**	**OCD**
Ratings	N-ISI	1.97 (1.40)	1.83 (1.50)	1.93 (1.35)	1.95 (1.15)	2.18 (1.59)
	N-CD	1.96 (1.16)	1.81 (1.25)	1.90 (1.13)	1.82 (0.91)	2.32 (1.28)
	P-ISI	2.87 (1.28)	2.83 (1.51)	2.90 (1.32)	3.03 (1.11)	2.71 (1.14)
	P-CD	3.40 (1.43)	3.17 (1.55)	3.33 (1.46)	3.72 (1.34)	3.40 (1.37)
	U-ISI	4.29 (1.64)	3.95 (2.02)	4.10 (1.13)	4.44 (1.33)	4.71 (1.75)
	U-CD	4.60 (1.67)	4.02 (1.93)	4.73 (1.45)	4.97 (1.33)	4.68 (1.77)
Startle (*t*)	N-ISI	46.40 (6.94)	46.36 (6.99)	46.72 (7.01)	46.57 (7.21)	45.85 (6.33)
	N-CD	46.09 (7.50)	46.76 (7.78)	46.24 (7.11)	45.85 (7.50)	45.42 (7.58)
	P-ISI	45.79 (7.26)	46.25 (7.19)	45.36 (7.82)	45.93 (6.55)	45.71 (7.47)
	P-CD	48.74 (8.69)	48.67 (8.64)	47.80 (8.16)	48.92 (8.47)	49.75 (9.55)
	U-ISI	50.04 (9.88)	49.68 (10.35)	49.93 (9.51)	50.41 (9.92)	50.22 (9.67)
	U-CD	54.59 (11.23)	54.01 (11.31)	54.20 (11.46)	55.49 (11.26)	54.64 (10.83)
SCR (μS)	N-ISI	0.03 (0.07)	0.03 (0.04)	0.02 (0.01)	0.03 (0.04)	0.05 (0.05)
	N-CD	0.03 (0.08)	0.03 (0.05)	0.02 (0.02)	0.03 (0.04)	0.04 (0.05)
	P-ISI	n.a.	n.a.	n.a.	n.a.	n.a.
	P-CD	0.06 (0.18)	0.05 (0.06)	0.05 (0.05)	0.06 (0.12)	0.07 (0.07)
	U-ISI	0.10 (0.17)	0.07 (0.06)	0.08 (0.05)	0.10 (0.05)	0.14 (0.13)
	U-CD	0.13 (0.24)	0.12 (0.12)	0.10 (0.08)	0.11 (0.11)	0.20 (0.20)
N1 (μV)	N-ISI	-13.00 (7.62)	-11.69 (8.25)	-14.38 (8.24)	-12.64 (6.70)	-13.38 (7.10)
	N-CD	-13.97 (7.33)	-12.88 (8.03)	-14.98 (8.39)	-13.53 (6.80)	-14.59 (5.74)
	P-ISI	-12.60 (6.99)	-11.57 (7.94)	-13.90 (7.45)	-12.55 (5.95)	-12.41 (6.36)
	P-CD	-15.04 (6.79)	-14.15 (8.15)	-15.62 (6.43)	-14.98 (6.10)	-15.50 (6.45)
	U-ISI	-15.29 (8.88)	-13.18 (9.52)	-18.16 (10.44)	-15.25 (5.88)	-14.64 (8.43)
	U-CD	-17.71 (8.22)	-16.42 (9.10)	-18.98 (8.50)	-17.07 (7.56)	-18.48 (7.56)
P3 (μV)	N-ISI	7.11 (4.90)	7.20 (5.72)	7.40 (5.36)	7.19 (4.29)	6.61 (4.06)
	N-CD	7.19 (5.04)	7.41 (5.47)	7.04 (5.34)	7.40 (4.91)	6.88 (4.51)
	P-ISI	6.08 (5.08)	6.69 (5.81)	6.03 (5.27)	5.44 (4.18)	6.11 (4.98)
	P-CD	5.12 (5.04)	5.79 (5.03)	5.42 (5.42)	5.01 (4.22)	4.13 (3.37)
	U-ISI	6.03 (5.39)	5.84 (5.62)	6.46 (6.03)	6.41 (4.94)	5.39 (4.97)
	U-CD	5.30 (5.21)	5.72 (5.44)	5.61 (5.40)	4.93 (4.81)	4.88 (5.28)

Note. Data from the NPU-threat test per outcome and group. Conditions include neutral (N), predictable threat (P), and unpredictable threat (U). Cues include the inter-stimulus interval (ISI) and countdown (CD). Groups include the control group, patients with specific phobia (PHOB), social anxiety disorder (SAD) and obsessive-compulsive disorder (OCD). Startle values are *t*-transformed.

Supplement B: Multilevel model for ratings

Table S2

Fixed Effects	*b*	*SE*	95% *CI*	*df*	*t*	*p*
Intercept	1.97	0.29	1.40 – 2.54	921	6.89	<.001
Predictable	0.73	0.35	0.05 – 1.41	921	2.10	.036
Unpredictable	1.73	0.40	0.96 - 2.51	921	4.38	<.001
PHOB	0.02	0.35	-0.66 - 0.70	921	0.06	.952
SAD	-0.35	0.36	-1.05 – 0.36	921	-0.97	.334
OCD	0.41	0.35	-0.29 – 1.10	921	1.15	.250
BDI	-0.02	0.03	-0.09 – 0.04	921	-0.69	.492
IUS	0.03	0.01	0.01 – 0.05	921	3.07	.002
MASQ	0.03	0.01	0.00 – 0.05	921	2.08	.038
Predictable × PHOB	0.43	0.41	-0.38 - 1.24	921	1.04	.299
Unpredictable ×PHOB	0.64	0.47	-0.29 – 1.57	921	1.35	.177
Predictable × SAD	0.72	0.42	-0.11 – 1.54	921	1.71	.087
Unpredictable × SAD	1.03	0.48	0.09 – 1.97	921	2.15	.032
Predictable × OCD	-0.29	0.42	-1.12 – 0.53	921	-0.70	.487
Unpredictable × OCD	0.39	0.48	-0.55 – 1.43	921	0.81	.416
Predictable × BDI	-0.06	0.04	-0.14 – 0.01	921	-1.60	.110
Unpredictable × BDI	-0.06	0.05	-0.15 – 0.03	921	-1.35	.177
PHOB × BDI	0.01	0.04	-0.07 – 0.08	921	0.24	.808
SAD × BDI	0.02	0.04	-0.06 – 0.09	921	0.44	.659
OCD × BDI	-0.03	0.04	-0.10 – 0.05	921	-0.71	.479
Predictable × PHOB × BDI	0.05	0.05	-0.04 – 0.14	921	1.14	.256
Unpredictable × PHOB × BDI	0.02	0.05	-0.08 – 0.13	921	0.47	.636
Predictable × SAD × BDI	0.07	0.04	-0.01 – 0.15	921	1.63	.103
Unpredictable × SAD × BDI	0.07	0.05	-0.03 – 0.17	921	1.45	.149
Predictable × OCD × BDI	0.13	0.04	0.04 – 0.21	921	2.91	.004
Unpredictable × OCD × BDI	0.12	0.05	0.02 – 0.21	921	2.43	.019
**Random Components**						
σ^2		0.58				
τ_{00}		0.95				
$\tau_{11\ \text{Predictable}}$		1.19				
$\tau_{11\ \text{Unpredictable}}$		1.73				
ICC		.70				
Marginal R^2		.390				
Conditional R^2		.819				
$N_{\text{participants}}$		158				
Observations		948				

Note. Groups include the unaffected control group, patients with specific phobia (PHOB), social anxiety disorder (SAD), and obsessive-compulsive disorder (OCD). Questionnaires include Beck Depression Inventory II (BDI), Intolerance of Uncertainty Scale-12 (IUS), and the Anxious Arousal subscale of the Mood and Anxiety Symptoms questionnaire (MASQ).
σ^2 = residual variance; τ_{00} = random intercept; ICC = intra class correlation.

Overall, participants showed increased ratings to predictable threat vs. no threat ($b = 0.89$, $SE = 0.15$, $t(143) = 6.17$, $p < .001$) and to unpredictable vs. no threat ($b = 2.18$, $SE = 0.17$, $t(143) = 13.19$, $p < .001$). Anxiety ratings to unpredictable threat were increased compared with predictable threat ($b = 1.28$, $SE = 0.12$, $t(143) = 10.37$, $p < .001$).

Supplement C: Multilevel model for skin conductance responses

Table S3

**Fixed Effects**	***b***	***SE***	**95% *CI***	***df***	***t***	***p***
Intercept	0.03	0.01	0.01 – 0.05	6051	2.76	.006
Predictable	0.01	0.01	-0.01 – 0.03	6051	1.34	.180
Unpredictable	0.05	0.01	0.03 – 0.07	6051	5.72	< .001
PHOB	-0.01	0.02	-0.04 – 0.02	6051	-0.75	.451
SAD	-0.00	0.02	-0.03 – 0.03	6051	-0.12	.902
OCD	0.01	0.02	-0.02 – 0.05	6051	0.83	.409
MASQ	0.00	0.00	-0.00 – 0.00	6051	0.13	.899
Predictable × PHOB	0.01	0.01	-0.01 – 0.04	6051	1.03	.304
Unpredictable × PHOB	0.02	0.01	-0.01 – 0.04	6051	1.43	.152
Predictable × SAD	0.02	0.01	-0.00 – 0.05	6051	1.65	.100
Unpredictable × SAD	0.03	0.01	0.01 – 0.05	6051	2.40	.016
Predictable × OCD	0.01	0.02	-0.02 – 0.04	6051	0.62	.533
Unpredictable × OCD	0.07	0.01	0.05 – 0.09	6051	5.63	< .001
Predictable × MASQ	-0.00	0.00	-0.00 – 0.00	6051	-1.35	.176
Unpredictable × MASQ	-0.00	0.00	-0.00 - -0.00	6051	-3.66	< .001
**Random Components**						
σ^2		.02				
τ_{00}		0.00				
ICC		.13				
Marginal R^2		.064				
Conditional R^2		0.187				
$N_{participants}$		145				
Observations		6068				

Note. Groups include the unaffected control group, patients with specific phobia (PHOB), social anxiety disorder (SAD), and obsessive-compulsive disorder (OCD). Questionnaires include the Anxious Arousal subscale of the Mood and Anxiety Symptoms questionnaire (MASQ).
σ^2 = residual variance; τ_{00} = random intercept; ICC = intra class correlation.

Follow-up contrasts across groups indicated that all groups showed larger SCRs in U than in N (b = 0.08, SE = 0.004, $t(5916)$ = 18.82, p < .001). Follow-up contrasts by group showed that controls displayed larger SCRs in U than in P (b = 0.04, SE = 0.010, t = 3.88, p < .001), whereas P did not differ significantly from N (b = 0.02, SE = 0.010, t = 1.81, p = .168). Similarly, the OCD

group showed larger SCR in U than in P ($b = 0.09$, $SE = 0.011$, $t = 8.71$, $p < .001$), while P did not differ from N ($b = 0.02$, $SE = 0.010$, $t = 1.97$, $p = .120$). By contrast, both the PHOB and SAD groups showed graded increases across all three conditions. In PHOB, SCRs were larger in P than in N ($b = 0.03$, $SE = 0.010$, $t = 2.95$, $p = .009$), in U than in N ($b = 0.07$, $SE = 0.008$, $t = 8.18$, $p < .001$), and in U than in P ($b = 0.04$, $SE = 0.010$, $t = 3.69$, $p < .001$). The same pattern emerged in SAD, with larger SCRs in P than in N ($b = 0.04$, $SE = 0.010$, $t = 3.66$, $p < .001$), in U than in N ($b = 0.07$, $SE = 0.008$, $t = 8.98$, $p < .001$), and in U than in P ($b = 0.04$, $SE = 0.010$, $t = 3.65$, $p < .001$).

The exploratory analysis including cue into the model yielded a main effect of cue ($F(1, 4726) = 15.75$, $p < .001$) and a condition × cue interaction ($F(1, 4724) = 20.69$, $p < .001$). Neither the cue × group interaction, ($F(3, 4726) = 1.49$, $p = .214$) nor the condition × cue × group interaction ($F(3, 4724) = 1.28$, $p = .281$) reached significance. Follow-up contrasts indicated that cue effects depended on condition: in the N condition, SCRs did not differ between ISI and CD ($b = 0.002$, $SE = 0.005$, $t(4725) = 0.44$, $p = .659$), whereas in the U condition, SCRs were larger during CD than ISI ($b = 0.034$, $SE = 0.006$, $t(4725) = 5.64$, $p < .001$).

Supplement D: Multilevel model for startle

Table S4

**Fixed Effects**	***b***	***SE***	**95% *CI***	***df***	***t***	***p***
Intercept	121.88	23.41	75.99 – 167.76	6433	5.21	<.001
Predictable	20.02	6.59	7.10 – 32.95	6433	3.04	.002
Unpredictable	37.92	6.50	25.18 – 50.67	6433	5.83	<.001
PHOB	-65.57	30.31	-124.98 – -6.16	6433	-2.16	.031
SAD	-66.28	35.44	-135.76 – 3.19	6433	-1.87	.061
OCD	-39.13	31.10	-100.10– 21.83	6433	-1.26	.208
IUS	3.19	2.42	-1.55 – 7.93	6433	1.32	.187
Predictable × PHOB	-23.99	8.59	-40.83 - -7.15	6433	-2.79	.005
Unpredictable × PHOB	-11.96	8.50	-28.62 – 4.70	6433	-1.41	.159
Predictable × SAD	-11.52	10.01	-31.13 – 8.10	6433	-1.15	.250
Unpredictable × SAD	-4.60	9.98	-24.16 – 14.96	6433	-0.46	.645
Predictable × OCD	-5.50	8.84	-22.82 – 11.82	6433	-0.62	.534
Unpredictable × OCD	8.35	8.79	-8.88 – 25.59	6433	0.95	.342
Predictable × IUS	2.31	0.69	0.99 – 3.64	6433	3.42	.001
Unpredictable × IUS	1.53	0.67	0.21 – 2.85	6433	2.27	.023
PHOB × IUS	-2.58	3.05	-8.56 – 3.40	6433	-0.85	.397
SAD × IUS	-3.19	3.48	-10.01 – 3.62	6433	-0.92	.359
OCD × IUS	-4.02	3.13	-10.16 – 2.12	6433	-1.28	.200
Predictable × PHOB × IUS	-3.06	0.86	-4.74 - -1.38	6433	-3.57	< .001
Unpredictable × PHOB × IUS	-1.37	0.85	-3.05 – 0.30	6433	-1.61	.108
Predictable × SAD × IUS	-2.73	0.97	-4.62 - -0.83	6433	-2.82	.005
Unpredictable × SAD × IUS	-2.16	0.97	-4.05 - -0.26	6433	-2.23	.026
Predictable × OCD × IUS	-2.08	0.89	-3.81 - -0.34	6433	-2.34	.019
Unpredictable × OCD × IUS	-0.95	0.89	-2.69 – 0.79	6433	-1.07	.286
**Random Components**						
σ^2		7691.57				
τ_{00}		13242.39				
ICC		.63				
Marginal R^2		.048				
Conditional R^2		.650				
$N_{participants}$		155				
Observations		6459				

Note. Groups include the unaffected control group, patients with specific phobia (PHOB), social anxiety disorder (SAD), and obsessive-compulsive disorder (OCD). The questionnaire included in the final model is the Intolerance of Uncertainty Scale-12 (IUS),
σ^2 = residual variance; τ_{00} = random intercept; ICC = intra class correlation.

Concerning task effects, the model yielded a main effect of condition ($F(2, 6289) = 66.97$, $p < .001$). Startle responses during condition U were increased compared with N ($b = 35.93$, $SE = 3.20$, $t(6289) = 11.21$, $p < .001$), and also compared with P (U>P, $b = 26.11$, $SE = 3.20$, $t(6289) = 8.15$, $p < .001$). Startle responses in condition P were increased compared with N ($b = 9.82$, $SE = 3.21$, $t(6289) = 3.06$, $p = .006$). Including a condition × cue interaction (i.e., countdown, interstimulus interval) into the model further specified task effects ($F(2, 6286) = 15.54$, $p < .001$). Specifically, startle responses in the interstimulus interval were significantly decreased compared with the countdown in conditions P ($b = -20.68$, $SE = 3.78$, $t(6286) = -5.48$, $p < .001$) and U ($b = -20.00$, $SE = 3.76$, $t(6286) = -5.32$, $p < .001$) but not in N ($b = 5.41$, $SE = 3.77$, $t(6286) = 1.43$, $p = .152$).

Supplement E: Multilevel model for N1

Table S5

**Fixed Effects**	***b***	***SE***	**95% *CI***	***df***	***t***	***p***
Intercept	-12.42	1.54	-15.43 - -9.40	916	-8.08	<.001
Predictable	-0.36	0.84	-2.00 – 1.28	916	-0.43	.669
Unpredictable	-3.16	0.84	-4.81 - -1.52	916	-3.78	<.001
PHOB	-2.64	1.89	-6.35 – 1.07	916	-1.40	.163
SAD	-0.61	2.12	-4.77 – 3.56	916	-0.29	.775
OCD	-1.62	2.06	-5.65 – 2.42	916	-0.79	.432
BDI	0.06	0.08	-0.09 – 0.21	916	0.79	.429
MASQ	-0.02	0.10	-0.21 – 0.17	916	-0.21	.832
PSWQ	-0.04	0.10	-0.25 – 0.16	916	-0.44	.664
Predictable × BDI	0.13	0.04	0.05 – 0.21	916	3.16	.002
Unpredictable × BDI	0.08	0.04	-0.01 – 0.16	916	1.83	.067
Predictable × MASQ	-0.13	0.05	-0.23 - -0.02	916	-2.35	.019
Unpredictable × MASQ	-0.03	0.05	-0.13 – 0.08	916	-0.55	.585
Predictable × PSWQ	-0.02	0.06	-0.13 – 0.09	916	-0.33	.742
Unpredictable × PSWQ	-0.10	0.06	-0.21 – 0.01	916	-1.80	.072
Predictable × PHOB	0.61	1.03	-1.40 – 2.63	916	0.60	.550
Unpredictable × PHOB	-0.50	1.03	-2.52 – 1.51	916	-0.49	.624
Predictable × SAD	-0.70	1.15	-2.97 – 1.56	916	-0.61	.543
Unpredictable × SAD	-0.96	1.15	-3.23 – 1.30	916	-0.84	.404
Predictable × OCD	0.62	1.12	-1.58 – 2.81	916	0.55	.583
Unpredictable × OCD	1.47	1.12	-0.73 – 3.66	916	1.31	.190
Predictable × PHOB × PSWQ	0.04	0.07	-0.09 – 0.18	916	0.62	.535
Unpredictable × PHOB × PSWQ	0.10	0.07	-0.04 – 0.24	916	1.43	.154
Predictable × SAD × PSWQ	0.01	0.09	-0.17 – 0.18	916	0.09	.931
Unpredictable × SAD × PSWQ	0.20	0.09	0.03 – 0.37	916	2.26	.024
Predictable × OCD × PSWQ	-0.06	0.08	-0.21 – 0.10	916	-0.73	.467
Unpredictable × OCD × PSWQ	-0.07	0.08	-0.22 – 0.08	916	-0.92	.356
**Random Components**						
σ^2		14.82				
τ_{00}		42.66				
ICC		.74				
Marginal R^2		.109				
Conditional R^2		.770				
$N_{participants}$		158				
Observations		948				

Note. Groups include the unaffected control group, patients with specific phobia (PHOB), social anxiety disorder (SAD), and obsessive-compulsive disorder (OCD). Questionnaires include Beck Depression Inventory II (BDI), the Anxious Arousal subscale of the Mood and Anxiety Symptoms questionnaire (MASQ), and the Penn State Worry Questionnaire (PSWQ).
σ^2 = residual variance; τ_{00} = random intercept; ICC = intra class correlation.

Concerning task effects, a main effect of condition emerged ($F(2, 770) = 44.64$, p <.001). The probe-locked N1 was increased (i.e., more negative) in the U condition relative to N (b = -3.17, SE = 0.37, $t(770) = -8.47$, p < .001) and in the U condition relative to the P condition (b = -2.94, SE = 0.37, $t(770) = -7.86$, p < .001). The N1 did not differ significantly in the N and P condition (b = 0.23, SE = 0.37, $t(770) = 0.60$, p = .818). Including the factor cue into the model led to a condition × cue interaction ($F(2, 767) = 4.13$, p = .016). While the probe-locked N1 was increased during the countdown compared to the interstimulus interval (b = -1.94, SE = 0.24, $t(767)$ = -8.12, p < .001), the cue-related enhancement was smaller in the N condition (countdown vs. interstimulus interval b = -0.97, SE = 0.41, $t(767) = -2.34$, p = .020), than in P (b = -2.44, SE = 0.41, $t(767) = -5.90$, p < .001) or U (b = -2.41, SE = 0.41, $t(767) = -5.83$, p < .001).

Supplement F: Multilevel model for P3

Table S6

**Fixed Effects**	***b***	***SE***	**95% *CI***	***df***	***t***	***p***
Intercept	7.15	0.37	6.42 – 7.87	156	19.29	<.001
Predictable	-1.55	0.21	-1.97 – -1.14	157	-7.34	<.001
Unpredictable	-1.48	0.23	-1.94 – -1.02	157	-6.32	<.001
MASQ	-0.12	0.04	-0.21 – -0.03	156	-2.71	.007
**Random Components**						
σ^2		5.25				
τ_{00}		19.08				
ICC		.79				
Marginal R^2		.064				
Conditional R^2		.799				
$N_{participants}$		158				
Observations		948				

Note. Mood and Anxiety Symptoms questionnaire (MASQ).
σ^2 = residual variance; τ_{00} = random intercept; ICC = intra class correlation.

Concerning task effects, the model yielded a main effect of condition, $F(2, 156) = 30.78$, $p < .001$. Relative to the neutral condition, P3 amplitudes were reduced in the predictable condition ($b = -1.55$, $SE = 0.21$, $t(156) = -7.32$, $p < .001$) and in the unpredictable condition ($b = -1.48$, $SE = 0.24$, $t(156) = -6.31$, $p < .001$).

Supplement G: Latent profile analysis model indices

Table S7

Profiles	LogLik	parameters	AIC	BIC	CAIC	SABIC	Entropy	prob_min	prob_max	n_min	n_max	BLRT_val	BLRT_p
1	-2428.87	24	4905.76	4976.86	5000.86	4900.92	1	1	1	1	1		
2	-2329.40	37	4732.82	4842.44	4879.44	4725.37	0.86	0.95	0.97	0.33	0.67	198.94	0.01
3	-2276.98	50	4653.96	4802.11	4852.10	4643.90	0.85	0.91	0.98	0.10	0.46	104.86	0.01
4	-2232.62	63	4591.26	4777.91	4840.91	4578.57	0.86	0.88	0.98	0.08	0.43	88.71	0.01
5	-2197.80	76	4547.61	4772.78	4848.78	4532.31	0.90	0.92	0.98	0.11	0.46	69.65	0.01

Note. LogLik = log-likelihood; AIC = Akaike information criterion; BIC = Bayesian information criterion; CAIC = consistent Akaike information criterion; SABIC = sample-size adjusted Bayesian information criterion; BLRT = bootstrapped likelihood ratio test; prob_min = lowest average posterior profile probability; prob_max = highest average posterior profile probability; n_min = smallest profile proportion; n_max = largest profile proportion.

Figure S1 Elbow plot for the latent profile analysis

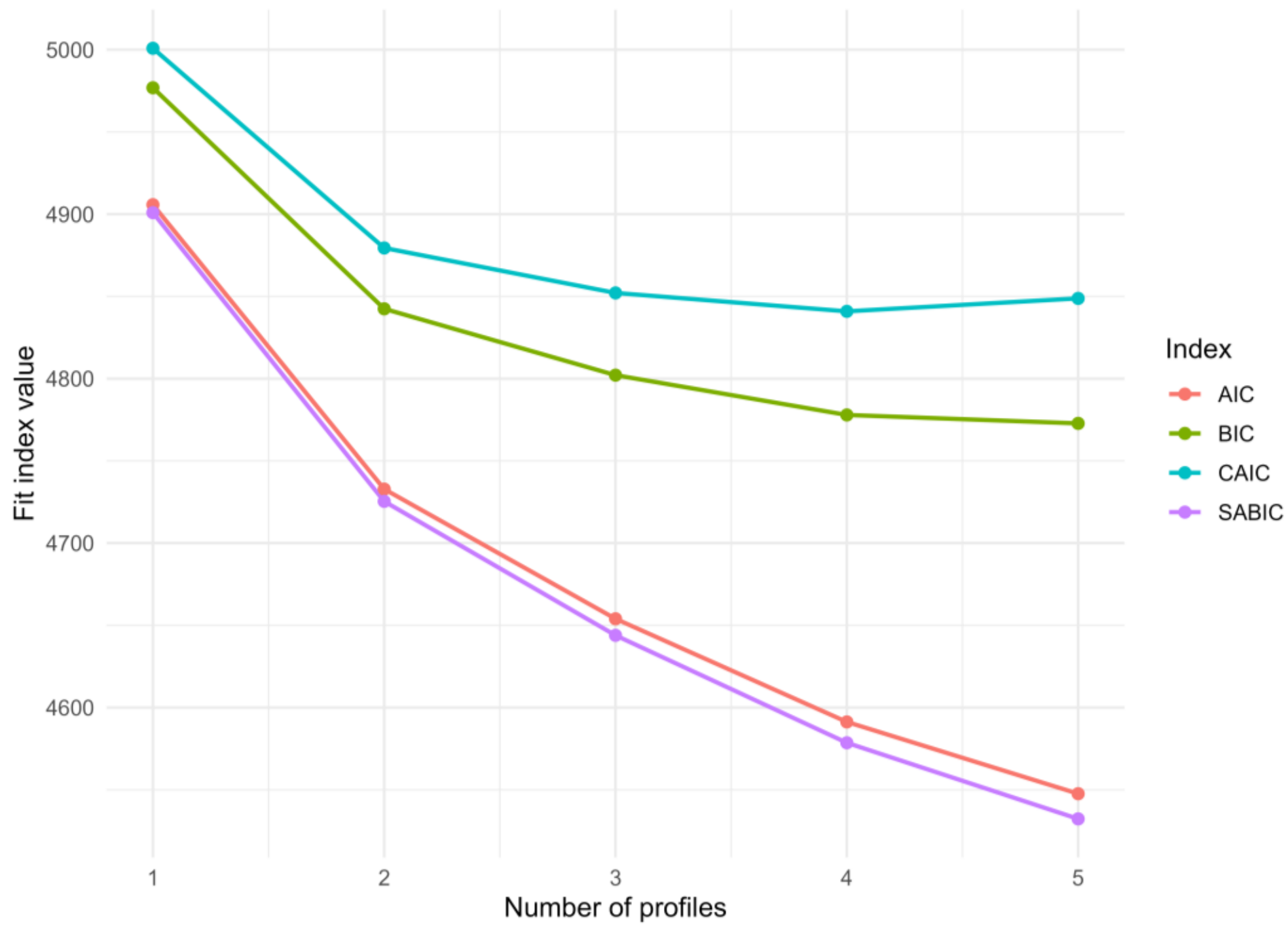

Note. AIC = Akaike Information Criterion BIC = Bayesian Information Criterion CAIC = Consistent Akaike Information Criterion SABIC = Sample-Size Adjusted Bayesian Information Criterion.

Supplement H: Physiological differences in latent profiles

Table S8

Omnibus tests from the profile × condition back-projection models.

Outcome	Condition	Profile	Profile × Condition
N1	*F*(2, 280) = 25.71, *p* < .001	*F*(2, 140) = 234.43, *p* < .001	*F*(4, 280) = 2.14, *p* = .076
P3	*F*(2, 280) = 24.73, *p* < .001	*F*(2, 140) = 3.38, *p* = .037	*F*(4, 280) = 0.14, *p* = .966
SCR	*F*(2, 280) = 150.98, *p* < .001	*F*(2, 140) = 2.68, *p* = .072	*F*(4, 280) = 4.34, *p* = .002
Startle	*F*(2, 420) = 77.72, *p* < .001	*F*(2, 420) = 2.27, *p* = .104	*F*(4, 420) = 1.49, *p* = .205

Note. Skin conductance response (SCR).

N1

For the probe-locked N1, the model yielded a main effect of condition, *F*(2, 280) = 25.71, *p* < .001, and a strong main effect of profile, *F*(2, 140) = 234.43, *p* < .001, whereas the Profile × Condition interaction did not reach significance, *F*(4, 280) = 2.14, *p* = .076. Thus, the retained profiles differed markedly in overall N1 amplitude, but not significantly in their NPU-specific modulation. Follow-up contrasts indicated that Profile 1 showed a more negative N1 in U than in N (*b* = -3.88, *SE* = 1.16, *p* = .003) and P (*b* = -3.62, *SE* = 1.16, *p* = .004), with no difference between N and P (*p* = .817). Profile 2 showed a graded pattern with increasingly negative amplitudes from N to P to U (all *p*s ≤ .004). Profile 3 also showed a more negative N1 in U than in N (*b* = -2.17, SE = 0.54, *p* < .001), whereas differences between P and the other two conditions were smaller and did not remain clearly significant after correction (*p*s ≥ .067). Overall, the N1 pattern suggests quantitative differences in early sensory processing across profiles rather than qualitatively distinct forms of condition sensitivity.

P3

For the probe-locked P3, there was a main effect of condition, $F(2, 280) = 24.73$, $p < .001$, and a main effect of profile, $F(2, 140) = 3.38$, $p = .037$, but no Profile × Condition interaction, $F(4, 280) = 0.14$, $p = .966$. Accordingly, all profiles showed lower P3 amplitudes in threat conditions than in N. In profile 1, both P and U were lower than N (ps ≤ .039), but P and U did not differ significantly ($p = .592$). The same pattern emerged in profiles 2 and 3, where P and U were both reduced relative to N (all ps < .001), and only profile 2 showed a modest difference between the two threat conditions, with slightly lower P3 amplitudes in P than in U ($b = 0.79$, $SE = 0.39$, $p = .046$). Thus, P3 suppression primarily tracked threat condition similarly across all profiles.

Skin conductance responses

For SCR, the model yielded a main effect of condition, $F(2, 280) = 150.98$, $p < .001$, whereas the main effect of profile was not significant, $F(2, 140) = 2.68$, $p = .072$. The profile × condition interaction was significant, $F(4, 280) = 4.34$, $p = .002$, indicating that the profiles differed in autonomic condition sensitivity. All three profiles showed the same ordinal pattern of N < P < U, with significant increases from N to P, N to U, and P to U within each profile (all ps ≤ .002). However, the magnitude of this increase differed across profiles. The increase from N to U was largest in profile 1 (Δ = .205) and profile 2 (Δ = .175), but smaller in profile 3 (Δ = .128), suggesting attenuated autonomic increase under threat, particularly unpredictable threat, in profile 3. Thus, SCR was the clearest physiological system in which the retained profiles differed in NPU modulation itself, not merely in overall response level.

Startle

For startle, there was a main effect of condition, $F(2, 420) = 77.72$, $p < .001$, but neither a main effect of profile, $F(2, 420) = 2.27$, $p = .104$, nor a Profile × Condition interaction, $F(4, 420) = 1.49$, $p = .205$. Across profiles, startle amplitudes were increased in U relative to N (all *ps* < .001) and in P relative to N (all *ps* ≤ .033). Differentiation between U and P was somewhat weaker in profile 1 ($b = 2.25$, $SE = 1.20$, $p = .062$) but clear in profiles 2 and 3 (both *ps* < .001). Thus, startle potentiation primarily reflected the NPU manipulation itself and showed little evidence of profile-specific modulation.

Figure S2 Estimated marginal means for profile × condition back-projection models

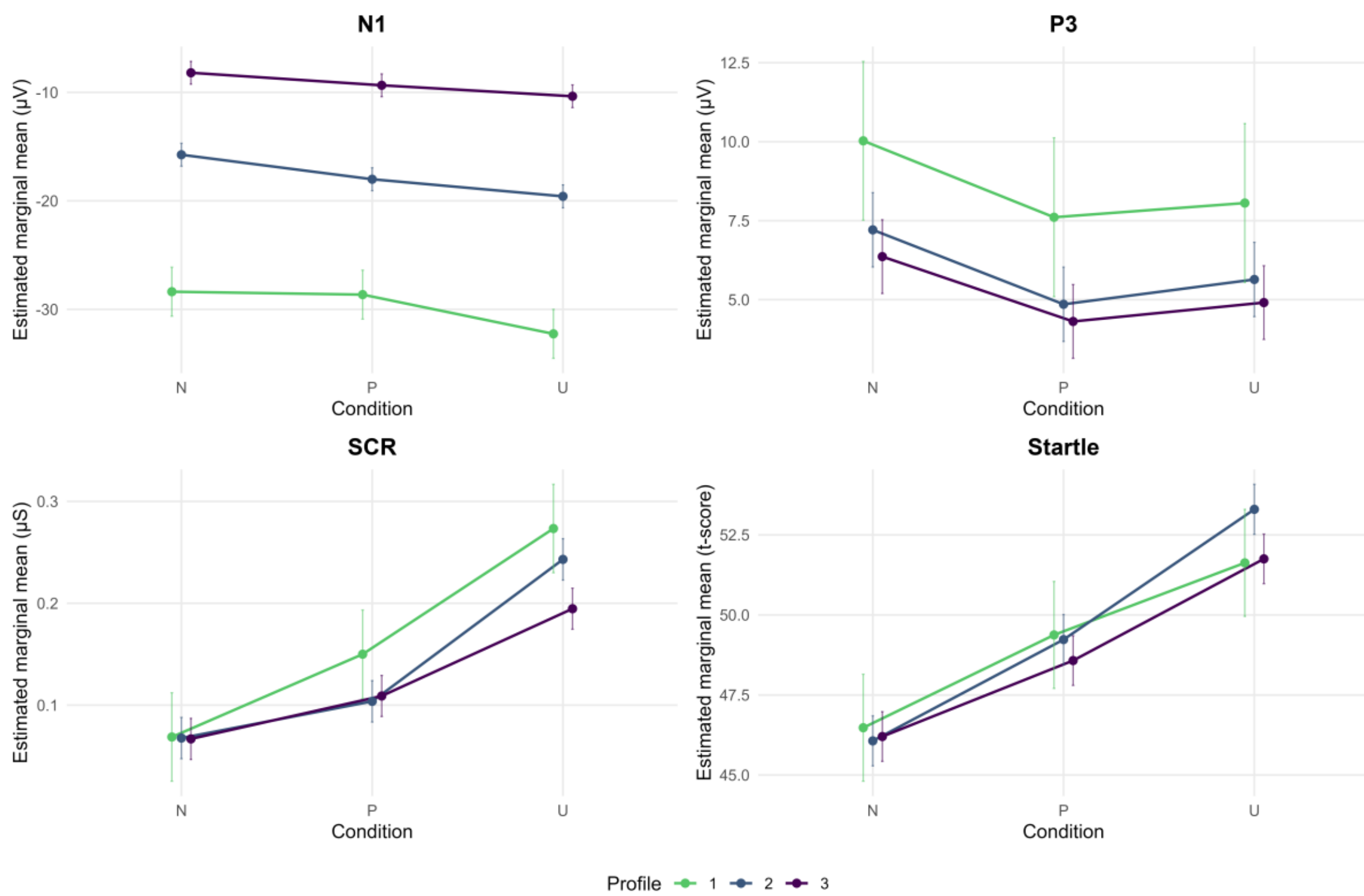

Note. Error bars represent +/- 1 standard error.

Supplement I: Clinical correlates of latent profiles

Figure S3 Clinical status across main latent profiles

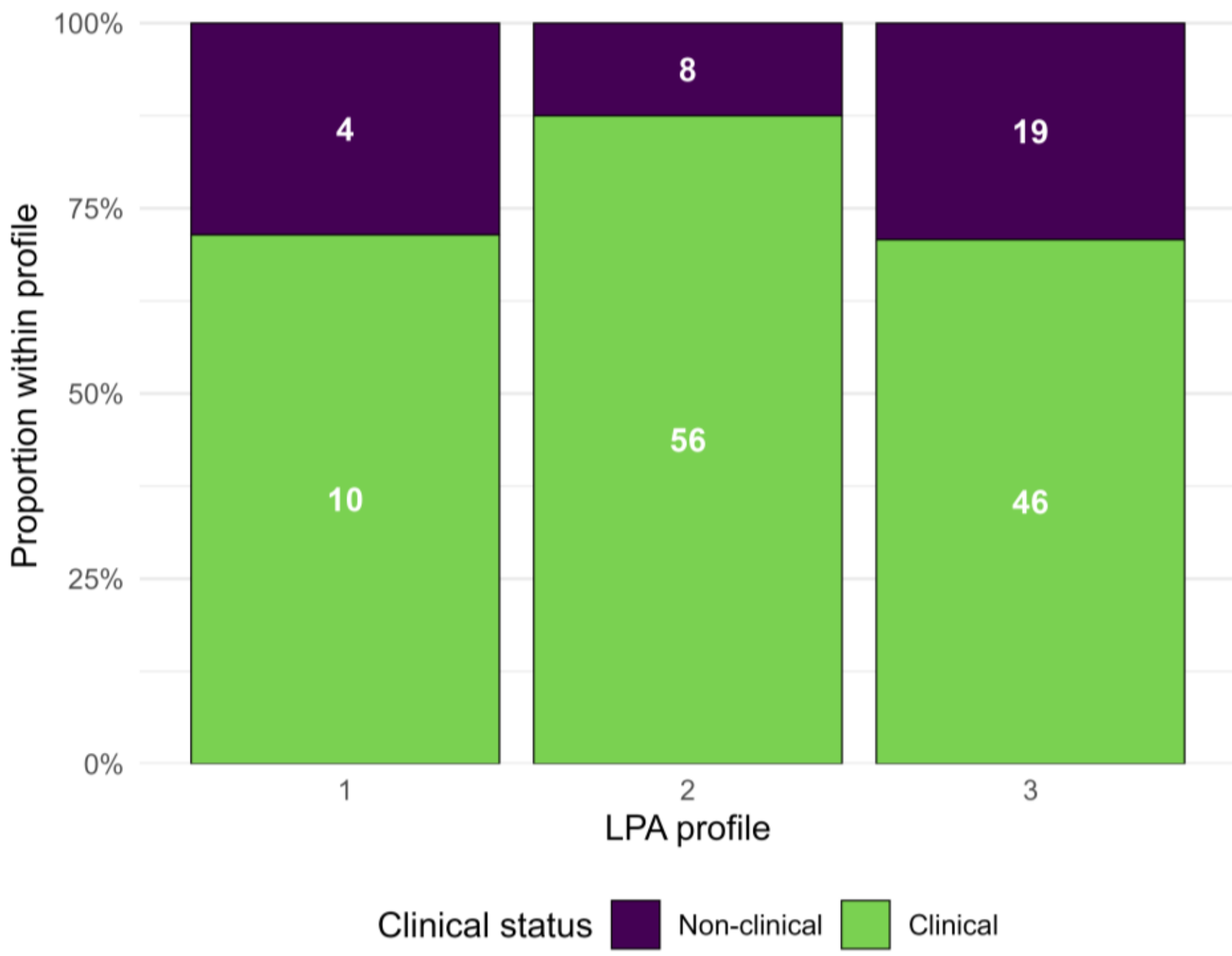

Note. Latent profile analysis (LPA) and counts of participants with any diagnosis within physiologically derived profiles.

Figure S4 Receiver operating characteristic for clinical status by physiological latent profile

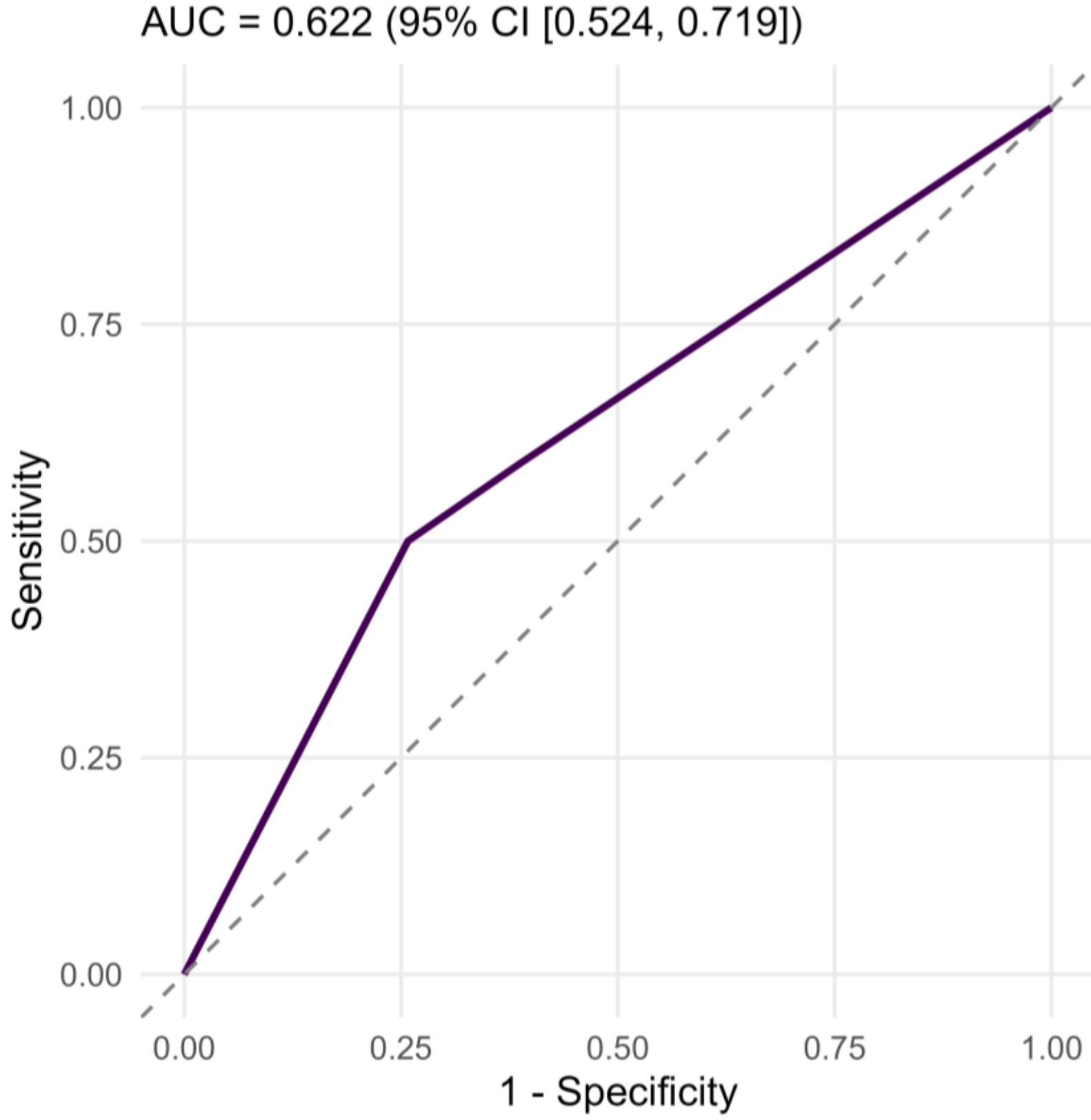

Table S9

Cellwise residual tests for diagnostic group by physiological profile

LPA profile	Group	Observed	Expected	Std. resid.	*p* (raw)	*p* (Holm)	Direction
2	SAD	24	16.56	2.86	.004	.051	overrepresented
2	HC	10	16.56	-2.52	.012	.129	underrepresented
3	SAD	12	16.82	-1.85	.065	.646	underrepresented
1	SAD	1	3.62	-1.69	.092	.828	underrepresented
1	HC	6	3.62	1.53	.127	.870	overrepresented
3	HC	21	16.82	1.60	.109	.870	overrepresented
2	PHOB	12	16.11	-1.59	.111	.870	underrepresented
3	PHOB	20	16.36	1.41	.159	.870	overrepresented
2	OCD	18	14.77	1.29	.197	.870	overrepresented
3	OCD	12	15.00	-1.11	.232	.870	underrepresented
1	PHOB	4	3.52	0.31	.758	.999	overrepresented
1	OCD	3	3.23	-0.15	.878	.999	underrepresented

Note. HC = unaffected controls; PHOB = specific phobia; SAD = social anxiety disorder; OCD = obsessive-compulsive disorder; LPA = latent profile analysis.

Supplement J: Prevalence of lifetime comorbid depressive disorders

Table S10

	**Full sample**	**HC**	**PHOB**	**SAD**	**OCD**
N	158	42	40	39	37
n lifetime	65	4	16	25	20
% lifetime	41.14	9.52	40.00	64.10	54.05

*Note. N*umber and percentage of participants with a current or lifetime history of a depressive disorder based on the SCID-5-CV, including current and remitted depressive episodes and recurrent depressive disorders. HC = unaffected controls; PHOB = specific phobia; SAD = social anxiety disorder; OCD = obsessive-compulsive disorder.
